# Huaxian formula alleviates nickel oxide nanoparticle-induced pulmonary fibrosis via PI3K/AKT signaling

**DOI:** 10.1038/s41598-025-01899-y

**Published:** 2025-05-22

**Authors:** Minmin Tian, Liruohan Feng, Mi Tian, Xiaodong Mu, Shi Bu, Jianfeng Liu, Jingyu Xie, Yujie Xie, Ling Hou, Guanghua Li

**Affiliations:** 1 School of Public Health, Ningxia Medical University, Yinchuan, 750004 Ningxia China; 2 School of Public Health, Shaanxi University of Chinese Medicine, Xianyang, 712046 Shaanxi China; 3 School of Basic Medicine, Ningxia Medical University, Yinchuan, 750004 Ningxia China

**Keywords:** Pulmonary fibrosis, Huaxian formula, PI3K/AKT pathway, Network Pharmacology, Experimental validation, Nickel oxide nanoparticles, Bioinformatics, Experimental organisms, Animal disease models, Respiratory tract diseases, Experimental models of disease

## Abstract

As a progressive fibrotic lung disorder with high mortality, pulmonary fibrosis (PF) suffers from inadequate treatment options. While the traditional Chinese medicine (TCM) formulation Huaxian Formula (HXF) demonstrates multi-target therapeutic potential against PF, the identity of its active components and their mechanistic basis of action require systematic investigation. To elucidate the therapeutic effects and pharmacological mechanisms of HXF in treating PF induced by nickel oxide nanoparticles (nano NiO), utilizing network pharmacology (NP), molecular docking, as well as in vivo and in vitro experiments. A comprehensive analysis of authoritative databases identified 121 active compounds, 202 potential therapeutic targets, and 1664 PF-related genes. Among these, 105 overlapping targets were found between HXF and PF. Gene Ontology (GO) and Kyoto Encyclopedia of Genes and Genomes (KEGG) analyses identified the PI3K/AKT signaling pathway as both a pivotal mechanism in PF pathogenesis and a primary target of HXF’s therapeutic effects. Molecular docking studies revealed that the six core compounds (quercetin, luteolin, kaempferol, β-sitosterol, isorhamnetin, and formononetin) of HXF exhibited strong binding affinity to proteins involved in the PI3K/AKT pathway. In the rat and A549 cell model, HXF treatment reduced collagen deposition and downregulated the expression of type I collagen (Col-I). Mechanistically, HXF inhibited the phosphorylation of PI3K and AKT. Collectively, these findings suggested that HXF alleviated PF by modulating the PI3K/AKT signaling pathway, providing valuable insights and methods for the development of TCM for PF.

**Supplementary Information:**

The online version contains supplementary material available at 10.1038/s41598-025-01899-y.

Nanomaterials encompass a diverse range of ultrafine particles with sizes between 1 and 100 nm. These materials consist of nano-sized microcrystals, including metallic, non-metallic, organic, inorganic, and biological powders^1^. Notably, nanomaterials exhibit remarkable durability and chemical stability^2^. Nickel oxide nanoparticles (nano NiO), a type of nanometal oxide, are widely utilized in medical healthcare, scientific research, and various industrial applications^3^. However, due to their unique physicochemical properties, nano NiO particles can penetrate deep into the alveoli and interstitial tissues, increasing the risk of pulmonary toxicity^4–6^. Animal studies have demonstrated that exposure to nano NiO is directly associated with the development of pulmonary fibrosis (PF). The underlying mechanism involves nano NiO-induced alveolar inflammation, which leads to collagen deposition and ultimately results in PF^5,7^. Our previous research has shown that nano NiO induces epithelial-mesenchymal transition and collagen deposition in A549 and BEAS-2B cells. This process involves TGF-β1-mediated activation of the PI3K/AKT and JNK/c-Jun signaling pathways^8,9^.

PF is a chronic progressive interstitial lung disease stimulated by continuous damage and excessive repair of alveolar epithelial cells^10^. A recent meta-analysis hass identified PF as an independent risk factor for lung cancer, with both high mortality and incidence rates worldwide^11^. Currently, antifibrotic agents such as nintedanib and pirfenidone have demonstrated efficacy in slowing disease progression; however, they fail to achieve disease stabilization or reversal. Additionally, these drugs are linked to a high incidence of adverse effects, particularly gastrointestinal and hepatic toxicity, further limiting their clinical utility^12,13^. Consequently, there is an urgent demand for the development of novel, safe and more effective therapeutic agents.

In Chinese medicine, PF is called “Feiwei”, mainly characterized by lung dryness-heat, lung qi deficiency, the consumption of body fluids, and involving blood stasis^14^. The Hua-Xian Formula (HXF), designed to address the pathogenesis and symptoms of PF, consists of seven ingredients: *Stephaniae tetrandrae radix* (FJ), *Rhizoma fagopyri cymosi* (JQM), *Platycladi cacumen* (CB), *Hedysarum Multijugum Maxim.* (HQ), *Platycodon grandiflorus* (JG), *Poria cocos (Schw.) Wolf.* (FL) and *Glycyrrhiza glabra L.* (GC)^15,16^. The therapeutic strategy of HXF aligns with core Chinese medicine doctrines in managing PF. HQ addresses the fundamental “intrinsic deficiency” by replenishing lung-spleen qi, thereby enhancing host defense mechanisms and tissue repair capacity^17,18^. Concurrently, JG and JQM collaboratively clear heat-toxin, resolve phlegm, and eliminate dampness, targeting key pathological factors in PF^16,19^. FG functions as a pulmonary decongestant by enhancing lymphatic drainage and capillary permeability, thereby reducing alveolar edema^20^. CB serves as a vascular stabilizer and bronchial relaxant, addressing hemoptysis and persistent cough through its bioactive flavonoids^16^. FL provides dual-modality support via aquaporin modulation for fluid homeostasis and immunomodulatory polysaccharides that reinforce the lung-spleen axis^17^. GC serves dual roles as a harmonizer to potentiate herb interactions and as a guide drug to enhance pulmonary targeting, while providing adjunctive antitussive and anti-inflammatory benefits^21^. This multi-target approach embodies the traditional Chinese medicine (TCM) principle of “simultaneous root-symptom treatment,” paralleling the mechanism of classical formula such as Buyang Huanwu Tang^22^. When the seven components are combined, these ingredients work synergistically to moisturize the lungs, detoxify the body, invigorate the spleen and qi, and promote the circulation of qi and blood^15,16^.

Our previous research indicated that HXF could inhibit the elevation of Type 1 collagen protein (Col-I) content in rat lung tissues induced by nano NiO, through the TGF-β1/Smads pathway^23^. Additionally, studies have suggested that HXF could counteract silica induced PF by modulating the TGF-β1/Smads pathway^15,16^. In clinical practice, HQ injection has demonstrated efficacy in treating patients with pulmonary tuberculosis and PF, potentially through the regulation of fibrosis-related factors and inflammatory cytokines^24,25^. Wang et al.^26^. found that HQ injection can be clinically used to treat PF induced by pneumoconiosis due to its potent anti-fibrotic effects. JQM is utilized in treating asthmatic bronchitis in infants and young children, exhibiting anti-inflammatory properties, promoting absorption of inflammatory exudates, and facilitating tissue repair and regeneration^27^. Tetrandrine, an alkaloid extracted from FG, has been shown to improve lung function in patients with pneumoconiosis. Administered alongside acetylcysteine, it effectively reduces inflammatory factor levels, alleviates cough symptoms, enhances treatment efficacy, and decreases the incidence of complications^28^. Moreover, a mixture of FL and GC can improve lung function, inhibit oxidative stress and inflammatory reactions, delay muscle atrophy. Consequently, this herbal mixture is utilized in the clinical management of chronic obstructive pulmonary disease associated with muscle atrophy^29^.

Although previous studies have shown that HXF can ameliorate PF, its precise mechanism of action remains unclear. Due to uncertainties regarding their composition and targets, TCM formulas are currently controversial in clinical applications. Network pharmacology (NP), a discipline, studies pharmacological effects by integrating systems biology and pharmacology. It focuses on the systematic analysis of interactions among drugs, targets, and disease-related biological pathways, aiming to understand the overall mechanism of drug action and predict potential side effects^30–32^. This approach provides an advanced technological mean to reveal the overall mechanisms of TCM treating diseases^33^. In addition, molecular docking analyses deliver crucial insights into the binding modes and stability of protein-drug complexes^34,35^.

Accordingly, we employed several high-precision, authoritative databases to identify the potential active constituents of HXF. Key compounds were then screened to forecast the core targets by combining them with disease databases. Functional enrichment analysis was performed to predict the primary mechanisms through which HXF exerts its effects on PF. Additionally, molecular docking was utilized to elucidate the ligand-target interactions at the molecular level between the active compounds and the primary targets. Furthermore, the rat PF model was established using nano-NiO in vivo. Masson staining of lung tissue was applied to observe the collagen deposition. The outcome indicators of PF and key pathway proteins were assessed to explore the efficacy and mechanisms of HXF. Finally, we developed an A549 cell model to study collagen deposition in vitro, further validating HXF’s therapeutic effects and underlying mechanisms in PF treatment. We were committed to determine the substance basis and mechanisms underlying the therapeutic potential of HXF. The content of our study was shown in Fig. 1.

**Fig. 1:**
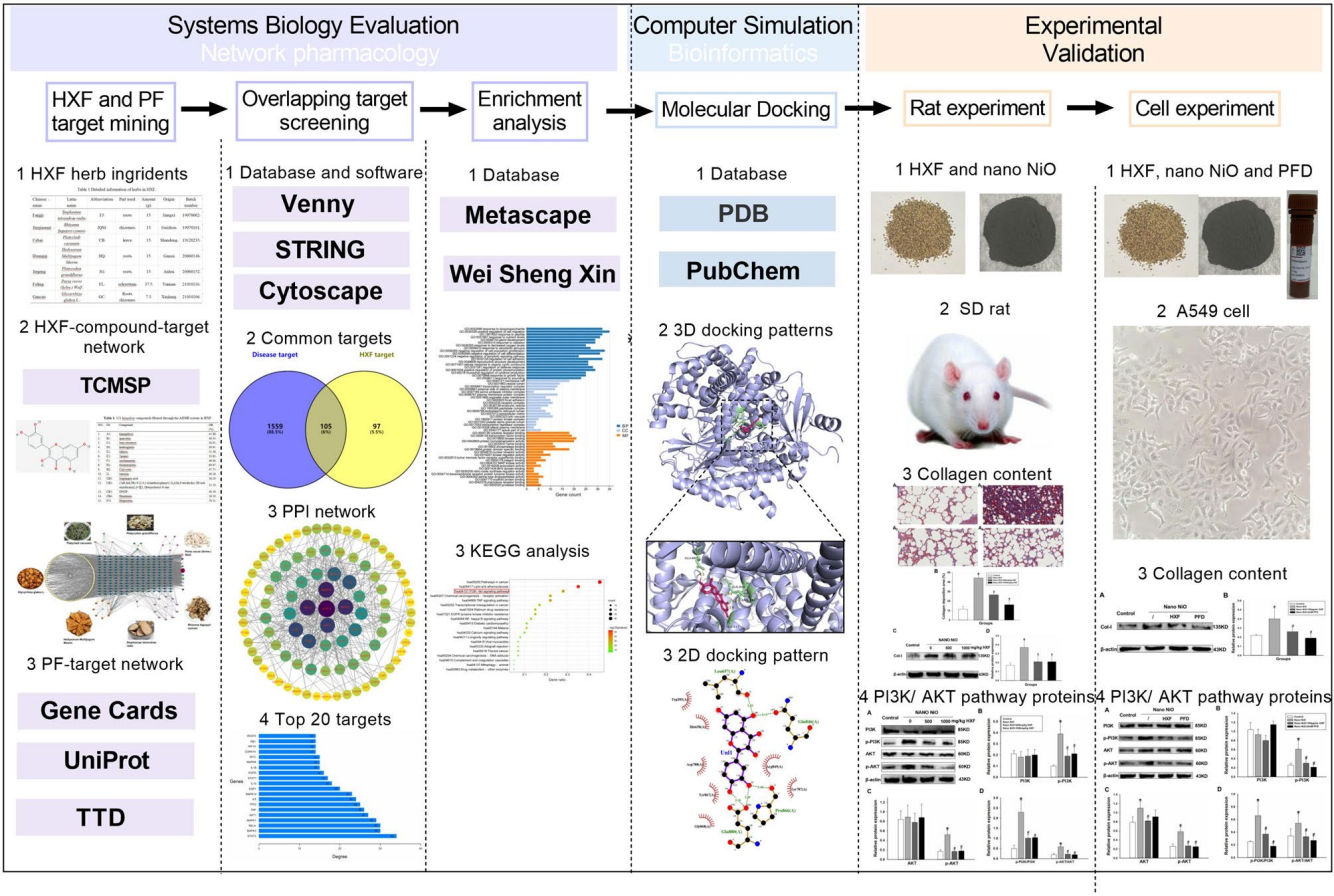
The graphical abstract of this study. This figure was generated using multiple computational tools including Cytoscape (v3.9.1, https://github.com/cytoscape/), AutoDock Vina (v1.5.7, https://autodock.scripps.edu/), LigPlot+ (v2.2.9, https://www.ebi.ac.uk/), PyMOL (v3.0.4, https://pymol.org/), and GraphPad Prism (v7.0, https://www.graphpad.com/).

## Results

### The NP analyses

#### Screening of the bioactive components and targets of HXF

**Fig. 2:**
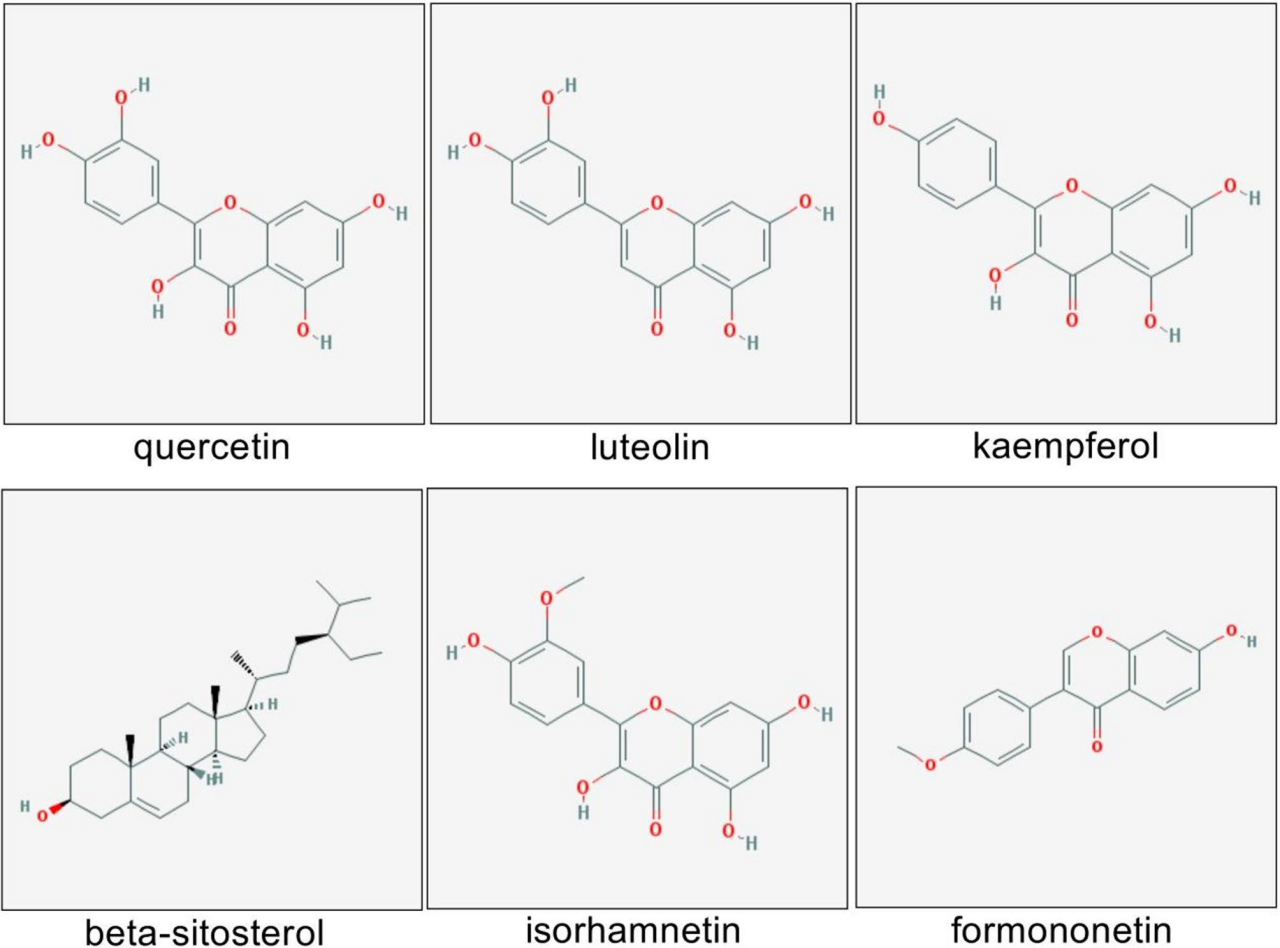
The molecular structure of the top six core components of HXF.

**Fig. 3:**
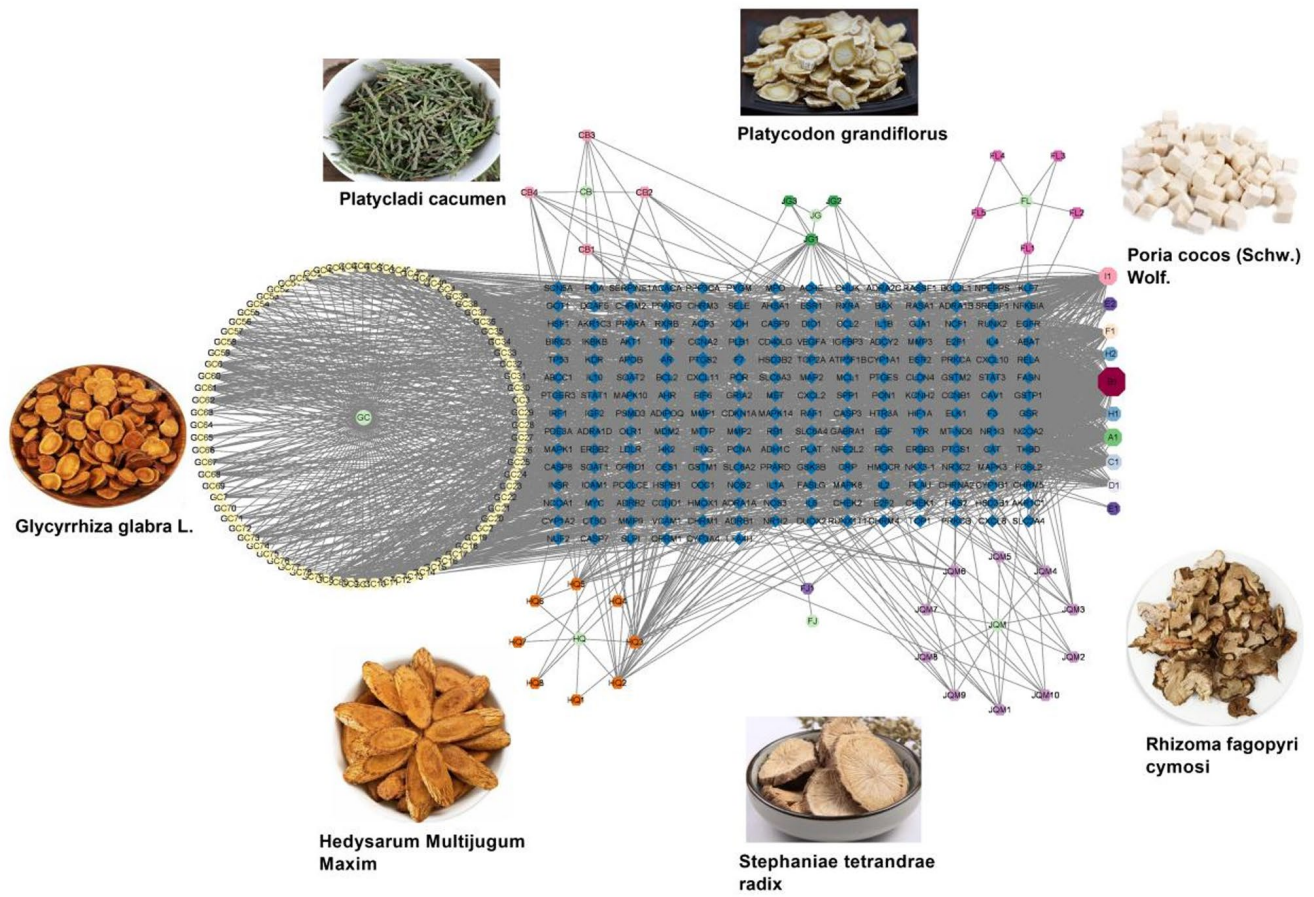
The active compound-targets of HXF. The hexagons represent the active compounds of herbs. The octagons on the right represent the shared components of herbs (A1: CB, GC and HQ; B1: CB, GC, HQ and JQM; C1: CB, FJ, GC and JQM; D1: FJ and HQ; E1, E2, H1, H2: GC and HQ; F1: GC, HQ and JQM; I1: JQM and JG).The blue quadrangles in the middle represent the targets. All edges represent the interaction between HXF and compounds or compounds and targets. This figure was generated using Cytoscape (v3.9.1, https://github.com/cytoscape/) and GraphPad Prism (v7.0, https://www.graphpad.com/).

According to ADME analysis, a total of 121 bioactive compounds from HXF were identified through the Traditional Chinese Medicine Systems Pharmacology (TCMSP) database, meeting the criteria of oral bioavailability (OB) ≥ 30% and drug likeness (DL) ≥ 0.18 (Supplementary Table S1). These compounds collectively interact with 202 potential targets, forming 4468 connections, with an average node degree of 13.50. Among these, 66 nodes had degree values exceeding the average. B1 (quercetin) exhibited the highest target interactions, followed by I1 (luteolin) and A1 (kaempferol). The top 6 core components were listed in Table 1, as well these structural formulas were listed in Fig. 2. Of the 202 targets, those with degree values above 70 include PTGS2 (120), ESR1 (87), AR (83), NCOA2 (77), NOS2 (77), and PTGS1 (76). The HXF-compound-target network was established by Cytoscape 3.9.1 (Fig. 3).

**Table 1:** The top 6 core components of HXF.

MOLID	Compound	Degree	molecular weight (g/moL)	CAS
MOL000098	Quercetin	492	302.23	73123-10-1
MOL000006	Luteolin	154	286.24	491-70-3
MOL000422	Kaempferol	144	286.24	520-18-3
MOL000358	Beta-sitosterol	78	414.70	83-46-5
MOL000354	Isorhamnetin	63	316.26	480-19-3
MOL000392	Formononetin	50	268.26	485-72-3

#### Identification of shared targets and construction of the PPI network

**Fig. 4:**
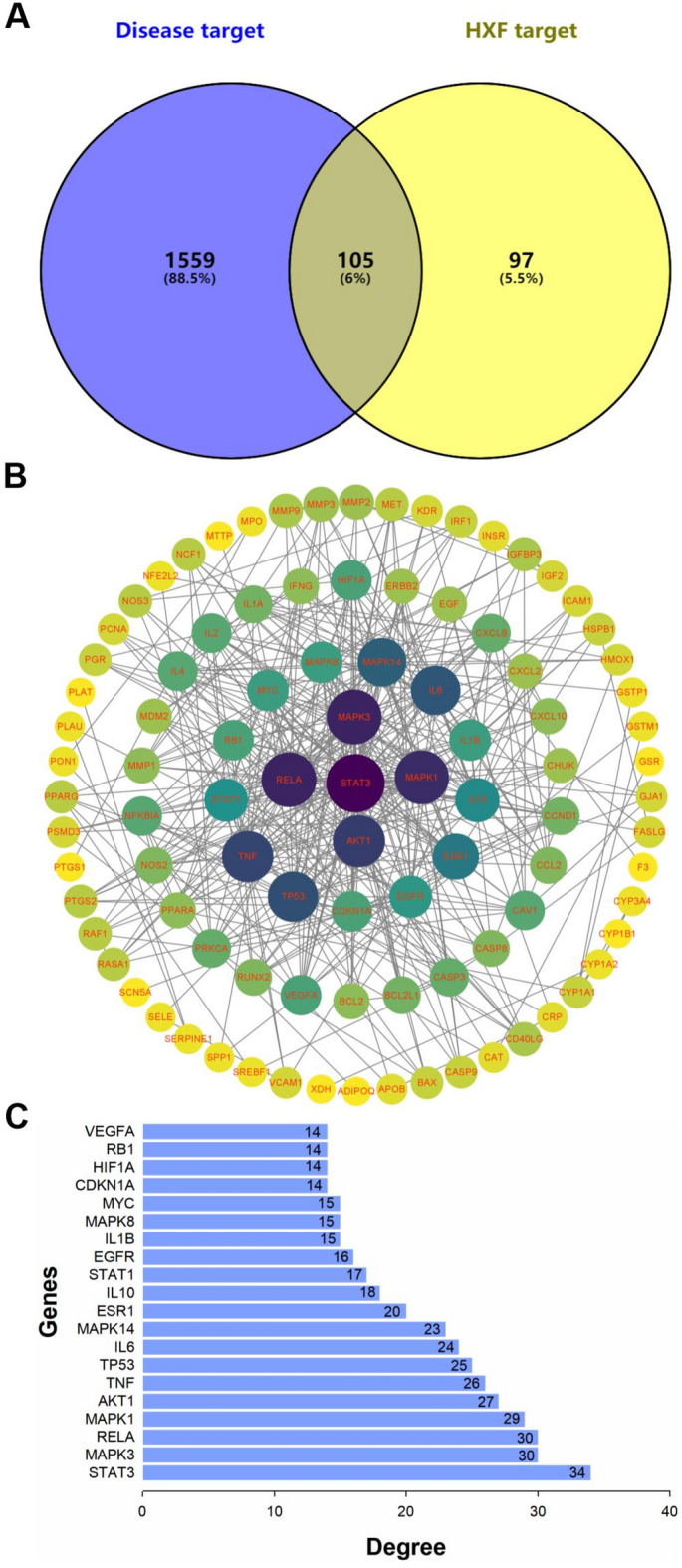
The protein-protein interaction (PPI) network of HXF targets for the treatment of PF. (**A**) The common targets of HXF and PF by venny2.1.0. (**B**) The detailed common targets of HXF and PF. Darker color and larger circle represent higher degree value. All edges represent the core targets interaction. (**C**) Top 20 core targets.

1664 PF targets were identified using authoritative databases. Among these, 105 targets overlapped with HXF, as shown in Fig. 4A, and were used to construct a PPI network visualized in Fig. 4B. The network consists of 105 nodes, 428 edges, with an average node degree of 8.15. Based on node degree, the top 20 key nodes were identified and are presented in Fig. 4C. The top five nodes in terms of degree values are STAT3, MAPK3, RELA, MAPK1, and AKT1.

#### Exploration of target pathways through gene ontology (GO) and Kyoto Encyclopedia of genes and geneomes (KEGG) analyses

**Fig. 5:**
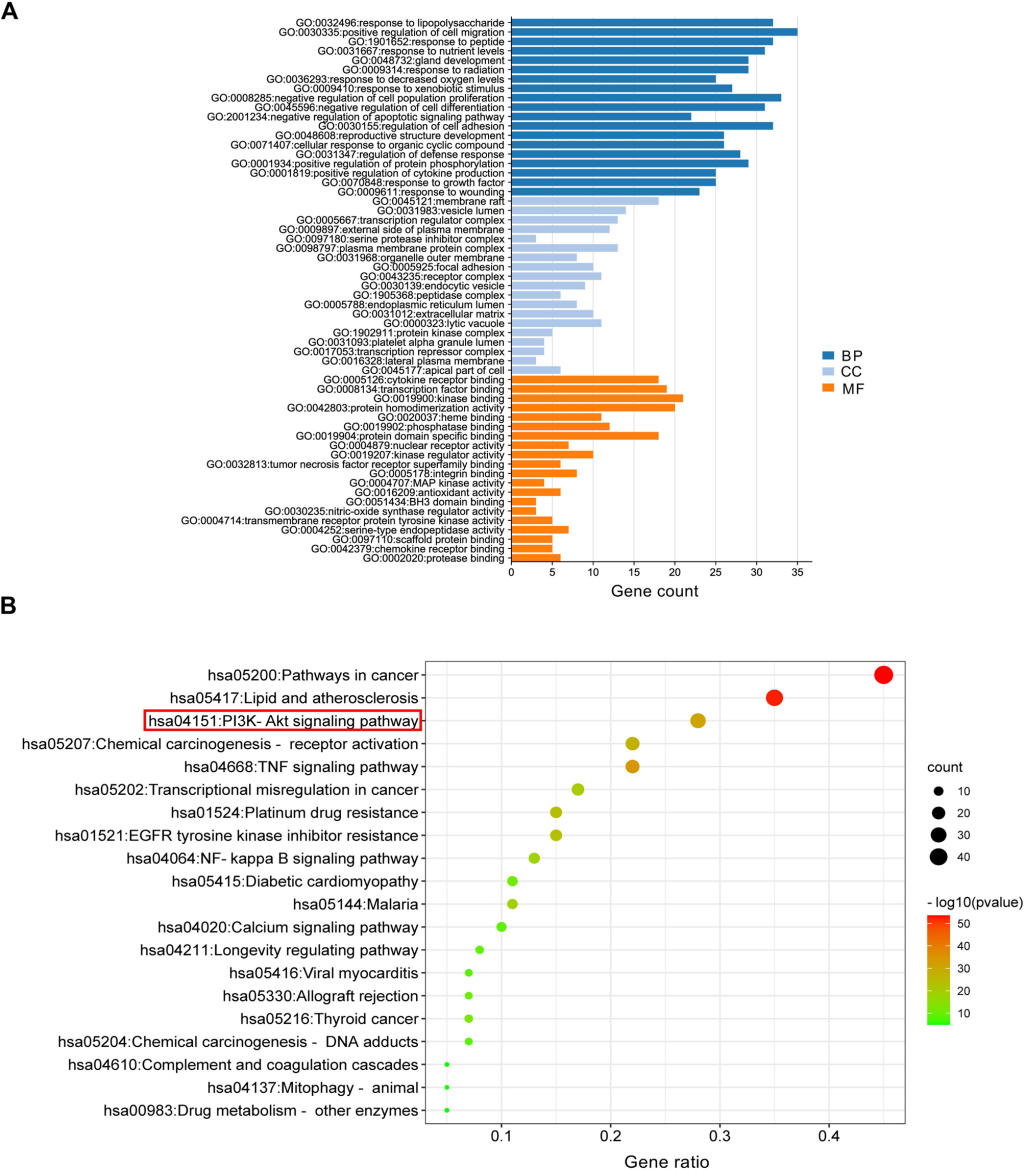
GO and KEGG enrichment analysis of 105 shared targets. (**A**) Go enrichment. (**B**) KEGG pathway analysis. Darker color and larger circle represent higher degree value. Large bubble represents the more genes involved, and redder bubble represents the more statistically significant. The red rectangular box on the left indicates that we will confirm it via subsequent experiments in this study. This figure was generated using KEGG (https://www.kegg.jp/kegg/kegg1.html) and Wei Sheng Xin (http://www.bioinformatics.com.cn/).

To clarify the biological functions and signaling pathways of 105 overlapping genes GO and KEGG enrichment analyses were conducted. The GO biological processes (BP) include “response to lipopolysaccharide”, “positive regulation of cell migration”, “response to peptide”, etc. These are associated with immune response, cellular signaling, and environmental responses. For cellular component (CC), enriched terms include “extracellular matrix”, “plasma membrane part”, and “platelet alpha granule lumen”. These results highlight the significant involvement of structural and signaling elements, particularly those related to membrane-associated and extracellular regions. In the molecular function (MF) category, “cytokine receptor binding”, “transcription factor binding”, and “protein homodimerization activity” were enriched notablely. These functions are crucial for receptor-ligand interactions, transcriptional regulation, and PPI within cellular processes (Fig. 5A). In addition, the KEGG pathway analysis HXF acting on PF mainly involves cancer pathway, lipid and atherosclerosis, PI3K-AKT signaling pathway”, chemical carcinogenesis-receptor activation, TNF signaling pathway and other signaling pathways (Fig. 5B).

### Analysis of molecular docking results

**Fig. 6:**
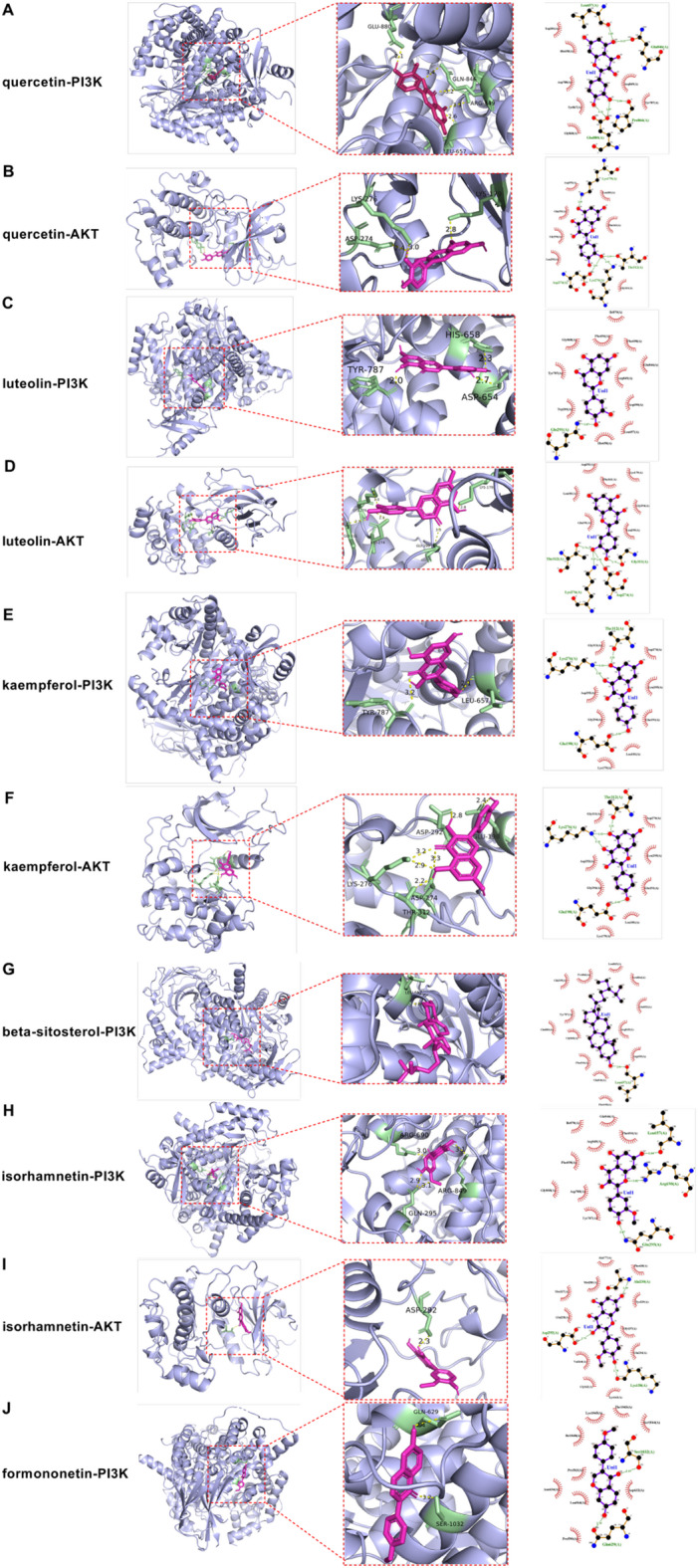
The 3D and 2D docking patterns and interactions of HXF with the targets in the PI3K/AKT signaling pathway. (**A-J**) The molecular docking quercetin with PI3K, quercetin with AKT, luteolin with PI3K, luteolin with AKT, kaempferol with PI3K, kaempferol with AKT, beta-sitosterol with PI3K, isorhamnetin with PI3K, isorhamnetin with AKT, and formononetin with AKT, respectively. The protein backbone is represented in light blue, amino acid residues interacting with ligands in pale green, and the drug molecules in light magenta. Yellow dashed lines indicate the distances between HXF components and amino acid residues in these 3D structures. This figure was generated using AutoDock Vina (v1.5.7, https://autodock.scripps.edu/), LigPlot+ (v2.2.9, https://www.ebi.ac.uk/), PyMOL (v3.0.4, https://pymol.org/), and GraphPad Prism (v7.0, https://www.graphpad.com/).

The six primary components of HXF—quercetin, luteolin, kaempferol, beta-sitosterol, isorhamnetin, and formononetin—were docked with protein molecules PI3K and AKT in the PI3K/AKT pathway. The binding energies, shown in Table 2, were all below zero, indicating that each ligand can spontaneously bind to its receptor. A lower binding energy reflects a more stable binding conformation. In this study, all 12 ligand-receptor pairs exhibited binding energies well below zero, suggesting stable interactions between the compounds and their targets. As shown in Fig. 6, the 3D and 2D docking results, visualized using PyMol, further illustrated these interactions. Notably, beta-sitosterol with AKT and formononetin with AKT did not display polar bonds, implying that their interactions may involve nonpolar forces instead (Supplementary Fig. S1).

**Table 2:** Binding energy values between the active compounds and the targets.

Ligand component	Receptor protein	Binding energy (kcal/mol)
Quercetin	PI3K	− 8.0
Quercetin	AKT	− 8.0
Luteolin	PI3K	− 8.3
Luteolin	AKT	− 8.1
Kaempferol	PI3K	− 8.4
Kaempferol	AKT	− 7.7
Beta− sitosterol	PI3K	− 7.9
Beta− sitosterol	AKT	− 7.9
Isorhamnetin	PI3K	− 8.0
Isorhamnetin	AKT	− 8.4
Formononetin	PI3K	− 7.7
Formononetin	AKT	− 7.8

### Animal experiment verification

#### Alleviation of HXF on nano NiO-induced PF in rats

**Fig. 7:**
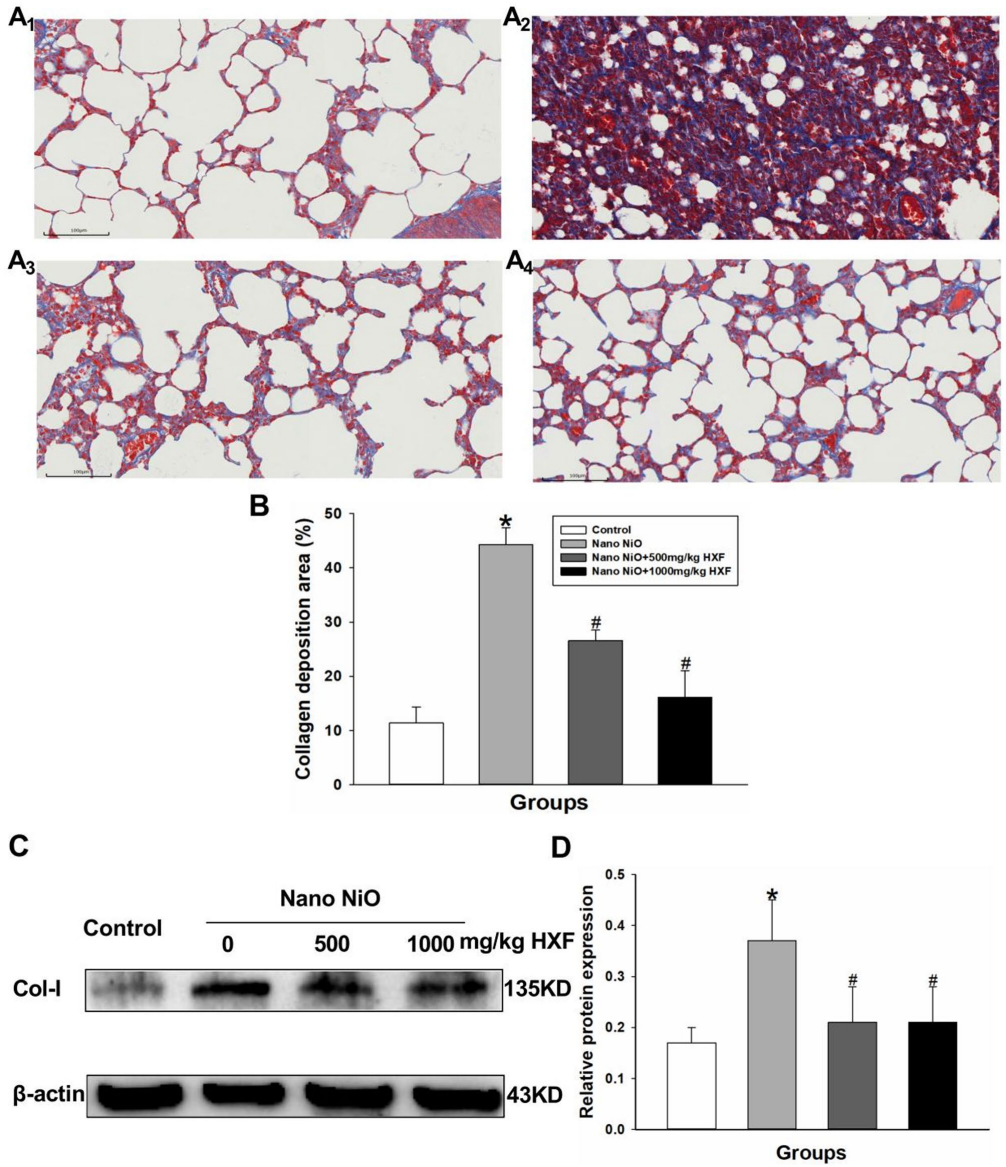
The HXF eased nano NiO-induced PF in the rats. (**A**_**1**_–**A**_**4**_) Collagen deposition in the control group, nano NiO group, nano NiO + 500 mg/kg HXF group, nano NiO + 1000 mg/kg HXF group by Masson staining, respectively (*n* = 3). (**B**) Semiquantitative analysis of collagen deposition. (**C**) Col-I protein bands. (**D**) Semiquantitative analysis of Col-I protein levels (*n* = 5). Values are expressed as mean ± SD. * The nano NiO group versus the control group, # nano NiO + HXF groups versus the nano NiO group (*p* < 0.05).

The anti-PF effect of HXF was evaluated in rats treated with nano NiO. Masson staining was performed to assess pathological changes in the lung tissue. The control group exhibited clear lung tissue with no signs of fibrosis. In contrast, the nano NiO-treated group showed altered alveolar size, structural damage, and significant collagen fiber deposition around the alveoli. In the nano NiO + 500 mg/kg HXF group, some alveoli appeared atrophic, and collagen fiber deposition was reduced, indicating an improvement in lung abnormalities induced by nano NiO. Notably, in the nano NiO + 1000 mg/kg HXF group, the lung tissue structure was largely intact, with a substantial reduction in blue-stained collagen fibers, approaching a nearly normal appearance (Fig. 7A_1_–A_4_ and B).

To evaluate the protective effects of HXF against nano NiO-induced PF, we analyzed key PF biomarkers in rats, with a specific focus on Col-I protein levels. As shown in Fig. 7C and D, Col-I protein expression was significantly higher in the nano NiO group compared to the control group (*p* < 0.05). However, treatment with HXF at doses of 500 mg/kg and 1000 mg/kg resulted in a marked reduction in Col-I protein expression (*p* < 0.05).

#### Regulation of HXF in PI3K/AKT signal pathway

**Fig. 8:**
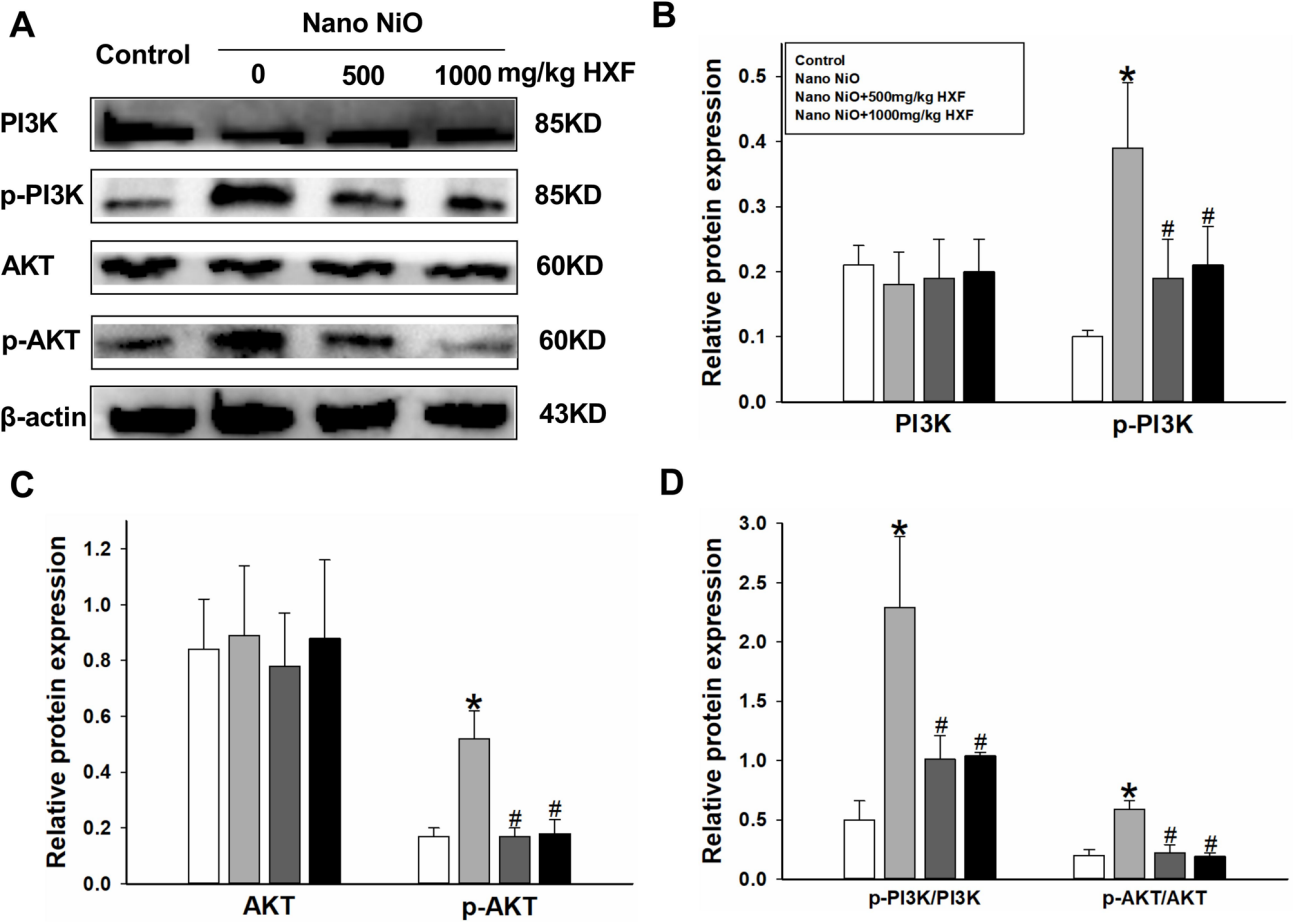
HXF inhibited PI3K/AKT transduction in Nano NiO administrated rats (*n* = 5). (**A**) Protein bands. (**B-D**) Semiquantitative analysis of protein levels. Values are expressed as mean ± SD. * The nano NiO group versus the control group, # nano NiO + HXF groups versus the nano NiO group (*p* < 0.05).

To confirm that HXF alleviates nano NiO-induced PF through the PI3K/AKT pathway, we examined the protein levels of PI3K, AKT, p-PI3K, and p-AKT. As illustrated in Fig. 8, the levels of p-PI3K, p-AKT, and their ratios (p-PI3K/PI3K and p-AKT/AKT) were significantly elevated in the nano NiO group compared to the control group (*p* < 0.05). In contrast, treatment with 500 and 1000 mg/kg HXF resulted in a significant downregulation of p-PI3K, p-AKT, p-PI3K/PI3K, and p-AKT/AKT levels compared to the nano NiO group (*p* < 0.05). Whereas, the protein expression levels of PI3K and AKT did not elicit any changes in each group (*p* > 0.05).

### Cell experiment verification

#### Evaluation of cell viability

**Fig. 9:**
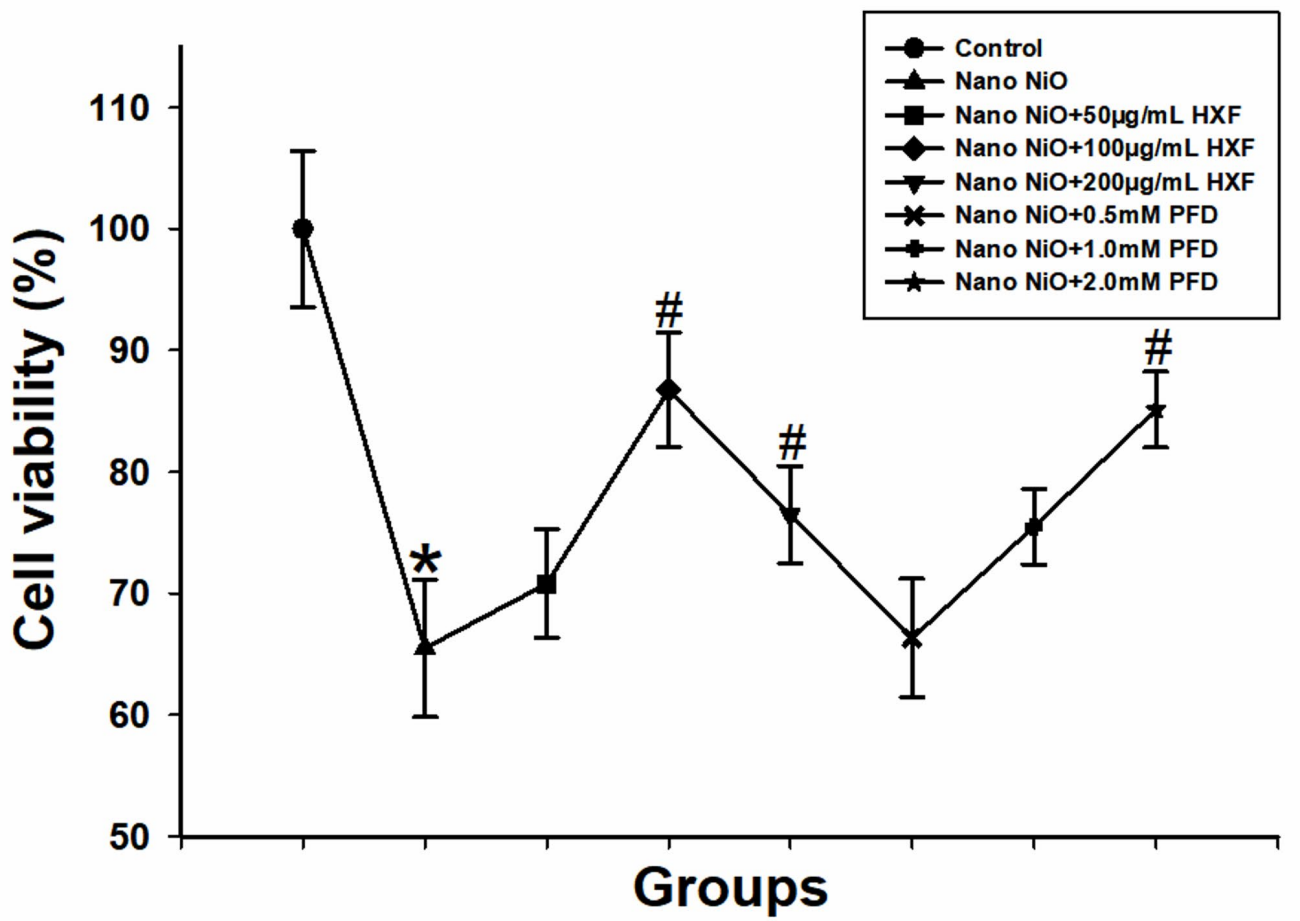
Cell viability in different treatment Groups in A549 cells (*n* = 5). Values are expressed as mean ± SD. * The nano NiO group versus the control group, # nano NiO combined with drug groups versus the nano NiO group (*p* < 0.05).

To determine the optimal doses of HXF and PFD, A549 cells were treated with various concentrations of HXF (0, 50, 100, 200, 400 µg/mL) or pirfenidone (PFD; 0, 0.5, 1, 2, 4 mM) for 48 h. The results displayed a significant reduction in cell viability compared to the control group after treatment with 400 µg/mL HXF and 4 mM PFD (*p* < 0.05; see Fig. S2 and Fig. S3). Based on the above results, cells were pretreated with nano NiO for 24 h and then treated with 50, 100, and 200 µg/mL HXF or 0.5, 1.0, and 2.0 mM PFD for 48 h. As depicted in Fig. 9, treatment with 100 µg/mL nano NiO resulted in significant cytotoxicity. However, 100 µg/mL HXF or 2 mM PFD effectively mitigated this toxicity. Therefore, these concentrations were selected for subsequent experiments.

#### Effect of HXF on nano NiO-induced collagen formation in A549 cells

**Fig. 10:**
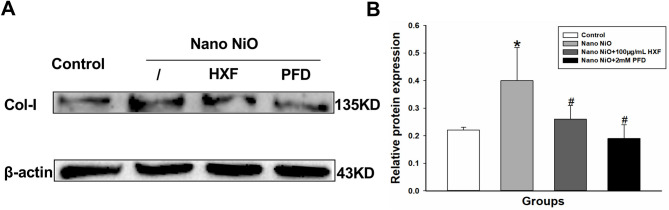
The HXF lessened nano NiO-induced collagen deposition in A549 cells. (**A**) Col-I protein bands. (**B**) Semiquantitative analysis of Col-I protein levels (*n* = 3). Values are expressed as mean ± SD. * The nano NiO group versus the control group, # nano NiO combined with drug groups versus the nano NiO group (*p* < 0.05).

To further validate the protective role of HXF against nano NiO-induced PF, we assessed Col-I protein expression in vitro. The results demonstrated that Col-I protein levels were markedly elevated in the nano NiO group than in the control group (*p* < 0.05). In contrast, treatment with 100 µg/mL HXF and 2mM PFD significantly attenuated Col-I protein expression (*p* < 0.05; Fig. 10).

#### Involvement of HXF in PI3K/AKT pathway regulation

**Fig. 11:**
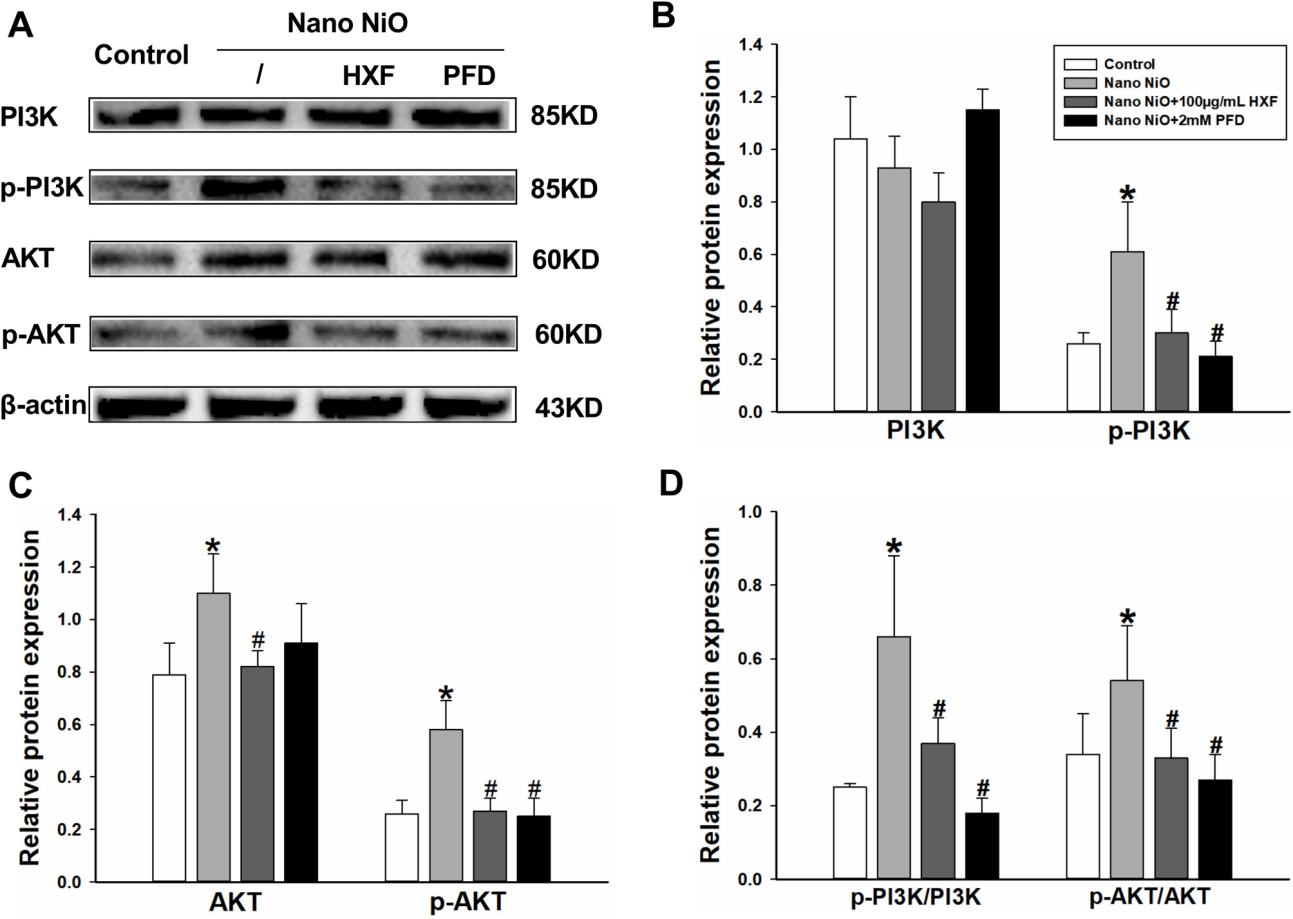
HXF suppressed PI3K/AKT signaling in A549 cells treated with Nano NiO (*n* = 3). (A) Protein bands. (B-D) Semiquantitative analysis of protein levels. Values are expressed as mean ± SD. * The nano NiO group versus the control group, # nano NiO combined with drug groups versus the nano NiO group (*p* < 0.05).

The expression levels of key proteins in the PI3K/AKT pathway were further assessed in A549 cells. In the Fig. 11, the levels of p-PI3K, AKT, p-AKT, and their respective ratios (p-PI3K/PI3K and p-AKT/AKT) were significantly higher in the nano NiO group compared to the control group (*p* < 0.05). Conversely, treatment with 100 µg/mL HXF notably lowered the levels of p-PI3K, AKT, p-AKT, and their ratios (p-PI3K/PI3K and p-AKT/AKT) compared to the nano NiO group (*p* < 0.05).

## Discussion

NiO Nanoparticles have attracted significant attention for their potential role in PF, a chronic, progressive lung disease involving excessive extracellular matrix deposition and fibrotic tissue formation^9^. In TCM theory, PF is interpreted as an imbalance of qi, blood, and fluid dynamics in pulmonary tissues, ultimately leading to fibrotic transformation and potentially malignant progression. To address this pathogenesis, the HXF formula was strategically developed as a combination of qi-tonifying (HQ and GC), heat-clearing (JQM and CB), and phlegm-resolving (FJ, JG and FL) herbs, each selected based on centuries of empirical TCM practice^15,16^. Meanwhile, modern pharmacological studies have begun to validate this traditional knowledge by identifying specific bioactive components within HXF that mechanistically combat fibrosis. For instance, Astragaloside IV from HQ has been shown to modulate TGF-β1/Smad pathway in a silica-induced rat model of PF, while quercetin (found in CB, GC, HQ and JQM) exhibits potent anti-inflammatory property^36,37^. This multi-herb formulation exemplifies the TCM principle of “synergistic multi-target therapy”, simultaneously addressing inflammation and fibroblast activation pathways implicated in nano NiO-induced PF. While preliminary studies have demonstrated the efficacy of HXF in alleviating PF via modulation of the TGF-β1/Smad pathway, the complete mechanistic understanding remains limited due to the inherent complexity of multi-component herbal formulations^15,23^. The present study aims to bridge this gap by integrating NP, molecular docking, and experimental validation in both cellular and animal models, offering a comprehensive elucidation of HXF’s anti-fibrotic mechanisms against nano NiO-triggered lung injury.

NP, employing computer-based simulations and network database retrieval, is utilized to identify key pharmacodynamic ingredients and targets. In this study, 121 core compounds and 202 corresponding key targets of HXF were identified. Among these, quercetin exhibited the highest number of interactions, suggesting that quercetin may be the primary active constituent of HXF for treating PF caused by nano NiO. This finding aligned with research by Hohmann et al.^38^. which demonstrated that quercetin reverses bleomycin-induced PF and downregulates pulmonary senescence markers in aged mice. Moreover, in this study’s PPI network, the top five degree-ranked common targets were STAT3, MAPK3, RELA, MAPK1, and AKT1. KEGG Pathway analysis showed significant enrichment in fibrosis-related pathways, with the PI3K/AKT signaling pathway emerging as one of the most prominent. GO (BP) analysis revealed that the common targets are primarily associated with responses to inorganic substances and exogenous stimuli, implicating their potential role in nano NiO-induced PF, with PI3K/AKT pathway-mediated cellular stress response. Furthermore, this pathway is related with regulating cell migration and apoptosis, processes that contribute to fibroblast persistence in fibrotic lesions. These findings underscore the potential central role of PI3K/AKT signaling in the therapeutic effects of HXF on PF.

The PI3K/AKT pathway serves as a master regulator of multiple cellular functions through its phosphorylation cascade^39^. PI3K catalyzes the transfer of G-phosphate from ATP to the D3 position of phosphatidylinositol, while AKT, a serine/threonine protein kinase, is activated in response to upstream PI3K. After activation, AKT signaling reaches various substrates with distinct functions^40^. Abnormal activity in this pathway is linked to multiple diseases, such as neurodegenerative diseases, stroke, diabetes, and cancer. Evidence indicates that overexpression of alpha-smooth muscle actin in lung fibrosis is associated with PI3K/AKT activation^41^. A clinical study demonstrated that p-AKT was increased 3-fold in alveolar macrophages of idiopathic PF patients compared with normal tissues^42^. Additionally, in clinical practice, the PI3K/AKT signaling pathway has been considered as a master regulator for PF. Inhibitors targeting this pathway are currently undergoing clinical evaluation and hold promise for the development of novel anti-fibrotic strategies^41^. Therefore, this study selected the PI3K/AKT signaling pathway in animal experiments to further validate HXF’s intervention effect on nano NiO-induced PF.

To validate the interactions between identified compounds and predicted targets, molecular docking analysis was conducted. Results suggested stable binding between certain compounds and verified targets. Quercetin, luteolin, kaempferol, beta-sitosterol, isorhamnetin, and formononetin displayed significant affinity for PI3K/AKT pathway proteins, thus highlighting these compounds as essential constituents of HXF in treating PF. These computational findings are further supported by existing experimental evidence, as multiple studies have demonstrated that quercetin, luteolin, kaempferol, and isorhamnetin can alleviate PF by modulating the PI3K/AKT signaling pathway^43–46^. The strong consistency between our molecular docking results and previously reported pharmacological data supports the anti-fibrotic potential of HXF.

In vivo experiments were performed to further explore potential mechanisms. Masson staining of lung tissue revealed that HXF treatment, especially at higher doses, effectively alleviated lung collagen deposition and alveolar shrinkage caused by nano NiO exposure. Collagen composition analysis from biopsy specimens of fibrotic patients revealed increased levels of Col-I and Col-III in early-stage fibrosis, whereas mature scar tissue predominantly consisted of Col-I^47^. Thus, Col-I was selected as an outcome indicator. Results showed nano NiO exposure led to increased Col-I protein levels, while HXF treatment significantly reduced Col-I, indicating that HXF can ameliorate nano NiO-induced PF. This finding aligned with Liu et al.^48^. which showed reduced collagen deposition in bleomycin-treated mice upon treatment with Qing-Xuan Granule. Notably, nano NiO significantly increased p-PI3K and p-AKT levels, while HXF inhibited their phosphorylation, suggesting that HXF may alleviate collagen overproduction by blocking the PI3K/AKT pathway. Interestingly, this mechanism is similar with the Jinshui Huanxian formula reported by Shao et al.^49^, which also targets PI3K/AKT signaling. Both formulas share GC and the bioactive compound isorhamnetin, indicating that these common components may underlie their anti-fibrotic effects and warrant further pharmacological investigation.

To further elucidate the underlying mechanisms, we conducted in vitro experiments. Cytotoxicity assays revealed that 100 µg/mL of HXF effectively mitigated the cytotoxic effects induced by nano NiO. Moreover, HXF significantly reduced collagen deposition and modulated the aberrant expression of key proteins in the PI3K/AKT pathway. As a positive control, 2 mM pirfenidone (PFD) was used to validate the anti-pulmonary fibrosis (PF) efficacy of HXF. It is noteworthy that HXF exhibited therapeutic effects and mechanisms of action comparable to those of PFD. These findings align with in vivo data, further supporting that HXF (a TCM) effectively modulates key proteins implicated in PF. This underscores its potential as a natural therapeutic agent or adjunct for clinical PF management.

## Conclusions

In this study, we investigated the therapeutic effects of HXF on nano NiO-induced PF and its underlying mechanisms by integrating NP prediction, molecular docking analysis, and experimental verification. Our results demonstrated that HXF could mitigate PF induced by nano NiO exposure. Quercetin, luteolin, kaempferol, beta-sitosterol, isorhamnetin, and formononetin were identified as the primary compounds in HXF responsible for this anti-fibrotic effect, acting through mechanisms associated with the PI3K/AKT signaling pathway. This study provided clues for potential clinical applications in treating PF.

## Materials and methods

### Analysis of NP

#### Screening of active components and presumed targets of HXF

The HXF comprises seven herbs: CB, FJ, FL, GC, HQ, JQM, and JG. To identify the primary bioactive components and their corresponding targets, we accessed the TCMSP database (https://old.tcmsp-e.com/index.php). We filtered the bioactive compounds of HXF based on the following criteria: OB ≥ 30% and DL ≥ 0.18. Additionally, we collected related protein targets for these bioactive compounds. Then the identified targets were standardized using the UniProt database (https://www.uniprot.org/). Finally, the Cytoscape3.9.1 was applied to construct the HXF-compound-target network.

#### Gathering of PF-associated targets

The related targets of PF from multiple databases, including GeneCards (https://www.genecards.org/), Therapeutic Target Database (TTD; http://db.idrblab.net/ttd/) and UniProt^50^. Besides, the common targets of HXF and PF were extracted via drawing Venn Diagram (https://bioinfogp.cnb.csic.es/tools/venny/).

#### PPI network analysis

The common targets were uploaded to the online STRING database (https://string-db.org/) with species restricted to Homo sapiens and confidence scores set to ≥ 0.9. Subsequently, the results were downloaded and utilized to generate the PPI network diagram using Cytoscape3.9.1. Eventually, the top 20 core nodes were identified based on their degree values and illustrated accordingly.

#### Enrichment analysis

To elucidate the gene functions and signaling pathways of HXF acting on PF, the common targets were submitted to the Metascape website (https://metascape.org/) for GO function and KEGG enrichment analyses (https://www.kegg.jp/kegg/kegg1.html). And results were visualized by the Bioinformatics website (http://www.bioinformatics.com.cn/).

### Molecular docking

AutoDock Vina1.5.7 was employed to conduct molecular docking simulations, aiming to predict both the binding affinity and conformation of key active ingredients with core protein targets. The active ingredients were initially obtained in sdf format from PubChem (https://pubchem.ncbi.nlm.nih.gov/) and converted to pdb format using Open Babel GUI. The 3D structure of each core protein was downloaded from the RCSB Protein Data Bank (PDB) (https://www.rcsb.org/), with water molecules and ligands removed using PyMOL3.0.4^51^. After that, Receptors (proteins) and ligands (active pharmaceutical ingredients) were imported into AutoDock Vina1.5.7 for docking. A grid box was configured, and the docking box was adjusted based on the molecular sizes. Docking outcomes were assessed through binding energy, with values < -4.5 kJ/mol indicating strong binding affinity between component and target^52^. Finally, PyMOL3.0.4 and LigPlot^+^2.2.9 were applied to visualize the docking results in 3D and 2D formats, respectively.

### Experimental study in vivo and in vitro

#### Chemicals and drugs

Nano NiO particles were obtained from ST-nano science and technology Co., Ltd (Shanghai, China). The characterization of these particles has been detailed in our previous study^53^. To ensure sterility, the nano NiO was treated at 120 °C for 30 min. The nano NiO suspension was then prepared using an ultrasonic homogenizer (Ningbo Scientz Biotechnology Co., Ltd, China).

The formulation consists of seven herbal granules, obtained from the affiliated hospital of Shaanxi University of Chinese Medicine. These granules were produced by licensed pharmaceutical manufacturers using a concentrated drying method. The quality ratio of FJ, JQM, CB, HQ, JG, FL and GC is 2: 2: 2: 2: 2: 5: 1^15,16,23^. The specific information of each component was listed in Table 3. HXF is transformed into granules through a series of processes, including grinding, extraction, concentration, and drying. This method can effectively extract the active ingredients from TCM formulas. Each step of the process conducts meticulous control of parameters to ensure the quality and efficacy of HXF.

**Table 3:** Detailed information of herbs in HXF.

Chinese name	Latin name	Abbreviation	Part used	Amount (g)	Origin	Batch number
Fangji	*Stephaniae tetrandrae radix*	FJ	Roots	15	Jiangxi	19,070,002
Jinqiaomai	*Rhizoma fagopyri cymosi*	JQM	Rhizomes	15	Guizhou	19,070,161
Cebai	*Platycladi cacumen*	CB	Leave	15	Shandong	19,120,235
Huangqi	*Hedysarum Multijugum Maxim*	HQ	Roots	15	Gansu	20,060,146
Jiegeng	*Platycodon grandiflorus*	JG	Roots	15	Anhui	20,060,152
Fuling	*Poria cocos (Schw.) Wolf.*	FL	Sclerotium	37.5	Yunnan	21,010,116
Gancao	*Glycyrrhiza glabra L.*	GC	Roots rhizomes	7.5	Xinjiang	21,010,106

#### Experimental animals

Thirty male Sprague-Dawley rats, weighing 200–220 g, were acquired from Chengdu Dossy Experimental Animals Co., Ltd (license: SCXK(Chuan)2020-030). The rats were housed in sterilized polypropylene cages with free access to food and water, under a 12-hour light/dark cycle at a temperature of 22–24 °C. They were acclimatized for one week prior to the start of the experiment. During the twenty-eight-day experimental period, the rats were fed a standard experimental diet. All animal procedures were performed in strict compliance with the Guide for the Care and Use of Laboratory Animals. The experimental protocols were reviewed and approved by the Animal Ethics Committee of Shaanxi University of Chinese Medicine (Approval No.: SUCMDL20210320001). Furthermore, all animal experiments were conducted in accordance with the ARRIVE guidelines.

#### Cell culture

A549 cells were provided by the Cancer Research Institute of Xi’an Jiaotong University (Shaanxi, China). The cells were cultured in Dulbecco’s Modified Eagle Medium (DMEM) supplemented with 10% fetal bovine serum (FBS) and 1% antibodies (100 units/mL of penicillin and 100 µg/mL of streptomycin), and were maintained at 37 °C in a humidified atmosphere containing 5% CO₂.

#### Study design

Rats were randomly divided into four groups: control, nano NiO, low-dose HXF, and high-dose HXF. The control group received intratracheal administration of physiological saline (0.9% NaCl), while rats in the other groups were administered a single intratracheal instillation of 1 mL nano NiO suspension (50 mg/mL) to induce lung fibrosis. Before the tracheal instillation procedure, the rats were anesthetized with an intraperitoneal injection of 50 mg/kg sodium pentobarbital. The following day, rats in the low-dose and high-dose HXF groups were orally gavaged with 500 mg/kg and 1000 mg/kg of HXF, respectively^15,16^. Rats in the control and nano NiO groups received distilled water instead. After 28 days of continuous treatment, the rats were anesthetized with 50 mg/kg sodium pentobarbital and euthanized by decapitation once fully anesthetized. Their lung tissues were then harvested for further analysis.

A549 cells underwent treatment with varying concentrations of HXF (0, 50, 100, 200, 400 µg/mL) or PFD (0, 0.5, 1, 2, 4 mM; TargetMol Chemicals Inc., Shanghai, China) for 48 h to perform CCK-8 assay^54,55^. HXF or PFD was dissolved in DMSO (Beijing Solarbio Science & Technology Co., Ltd, China), with the final concentration of DMSO in the solution being 0.1%. According to these preliminary results, cells were pretreated with 100 µg/mL nano NiO for 24 hours^56^, followed by treatment with 50, 100, and 200 µg/mL HXF or 0.5, 1.0, and 2.0 mM PFD for an additional 48 h to assess cell viability. From these findings, 100 µg/mL HXF and 2.0 mM PFD were selected for further analysis of the protein expression levels of Col-I, PI3K, AKT, p-PI3K, and p-AKT.

#### Histopathological examination

Three rats were randomly selected from each group, and the frontal lobes of the right lung were fixed in 4% paraformaldehyde at room temperature and embedded in paraffin. The tissues were sectioned into 4 μm slices and stained with Masson’s trichrome. These sections were then scanned using the SQS-40R Slide Scanning System (40X; Shengqiang Technology Co., Ltd, China). Randomly selected 3 regions from each slide and performed quantitative analysis on collagen content (blue staining) by ImageJ and visualize it using GraphPad Prism 7.0. Collagen deposition area (%) = Blue staining area / Total view area × 100%.

#### CCK-8 assay

Cell viability was measured via the CCK-8 Cell Proliferation Detection Kit (TargetMol Chemicals Inc., Shanghai, China) in triplicate. A549 cells were seeded into 96-well plates at a density of 5000 cells per well and incubated. Following incubation, 10 µL of CCK-8 reagent was added to each well, and the plates were incubated at 37 °C for 1–4 h in the incubator. The absorbance of each well was then assessed at 450 nm using a microplate reader (KAIAO technology, China).

#### Western blot

The 80 mg middle lobes of right lung tissues were collected from each rat to assess protein content. The tissue samples were homogenized in lysis buffer containing 800 µL RIPA, 8 µL PMSF and 8 µL Phosphatase Inhibitor. Total protein levels were determined using a protein assay kit (Boster Biological Technology Co. Ltd., China). Equal amounts of protein were separated by SDS-PAGE and transferred to PVDF membranes. Membranes were blocked with 5% skim milk for 4 h at room temperature, followed by overnight incubation at 4 °C with primary antibodies at a 1:500 dilution. Primary antibodies included β-actin (Signalway Antibody, USA), Col-I, PI3K, p-PI3K, AKT, and p-AKT (Immunoway, USA). After membranes were incubated with goat anti-rabbit secondary antibodies (1:5000 dilution) for 2 h. Protein detection was performed using an enhanced chemiluminescence system (Thermo Fisher Scientific, USA) and visualized on a Molecular Imager ChemiDoc XRS System (Bio-Rad, USA). Quantitative analysis of protein bands was conducted using IPP software (Media Cybernetics Inc., China), with protein expression normalized to β-actin.

#### Statistical analysis

The data analysis was performed using SPSS26.0 and the results were expressed as mean ± standard deviation (SD). The groups were compared by one-way ANOVA. Post-hoc comparisons were performed using LSD if variances were equal; otherwise, Dunnett’s T3 was applied. A p-value less than 0.05 was considered statistically significant.

## Electronic supplementary material

Below is the link to the electronic supplementary material.

Supplementary Material 1

Supplementary Material 2

## Data Availability

The datasets used and/or analysed during the current study available from the corresponding author on reasonable request.

## Abbreviations

HXF: Huaxian formula
TCM: Traditional Chinese Medicine
PF: Pulmonary fibrosis
nano NiO: Nickel oxide nanoparticles
NP: Network pharmacology
TCMSP: Traditional Chinese Medicine Systems Pharmacology
TTD: Therapeutic target database
PPI: Protein–protein interaction
GO: Gene ontology
KEGG: Kyoto encyclopedia of genes and genomes
Col-I: Type I collagen
FJ: *Stephaniae tetrandrae radix*
JQM: *Rhizoma fagopyri cymosi*
CB: *Platycladi cacumen*
HQ: *Hedysarum Multijugum Maxim.*
JG: *Platycodon grandiflorus*
FL: *Poria cocos (Schw.) Wolf*
GC: *Glycyrrhiza glabra L.*
OB: Oral bioavailability
DL: Drug likeness
ADME: Absorption, distribution, metabolism, and excretion
PDB: RCSB protein data bank
SD: Standard deviation
PFD: Pirfenidone
