# Supplementary material for: Huaxian formula alleviates nickel oxide nanoparticle-induced pulmonary fibrosis via PI3K/AKT signaling
Source: Sci Rep. 2025 May 22;15:17862. doi: 10.1038/s41598-025-01899-y (PMC12098777; doi:10.1038/s41598-025-01899-y)

IB

Protein Name: β-actin

Brand: Signalway Antibody

Observed Band: 43KD

Catalog No.: 21338-1

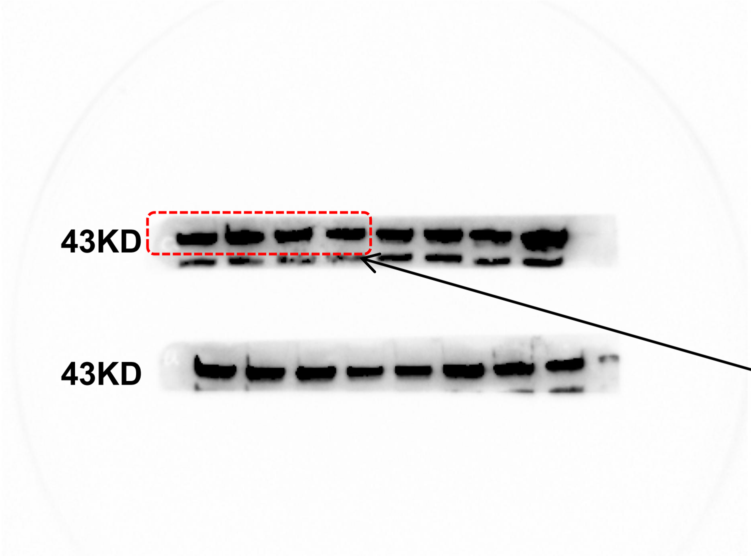

Figure 8

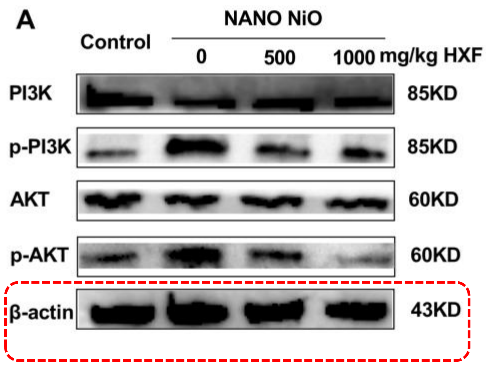

From left to right: control group, nano NiO group, nano NiO + 500 mg/kg HXF, and nano NiO + 1000 mg/kg HXF.

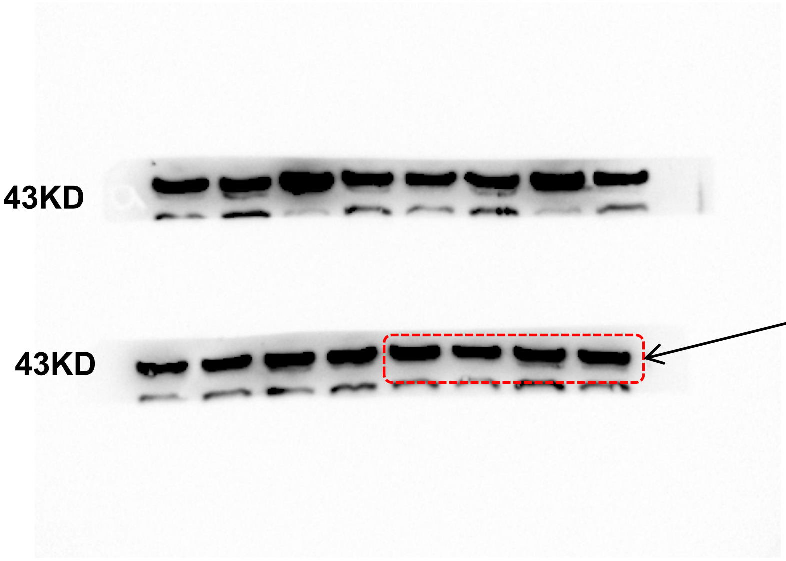

Figure 7

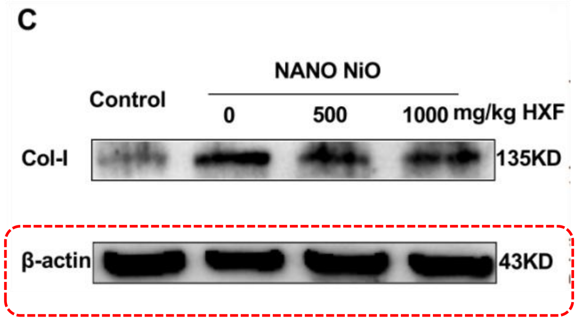

IB

Protein Name: Col-I

Brand: Immunoway

Observed Band: 135KD

Catalog No.: YT5769

From left to right: control group, nano NiO group, nano NiO + 500 mg/kg HXF, and nano NiO + 1000 mg/kg HXF. Each target protein was tested at least three times.

Figure 7

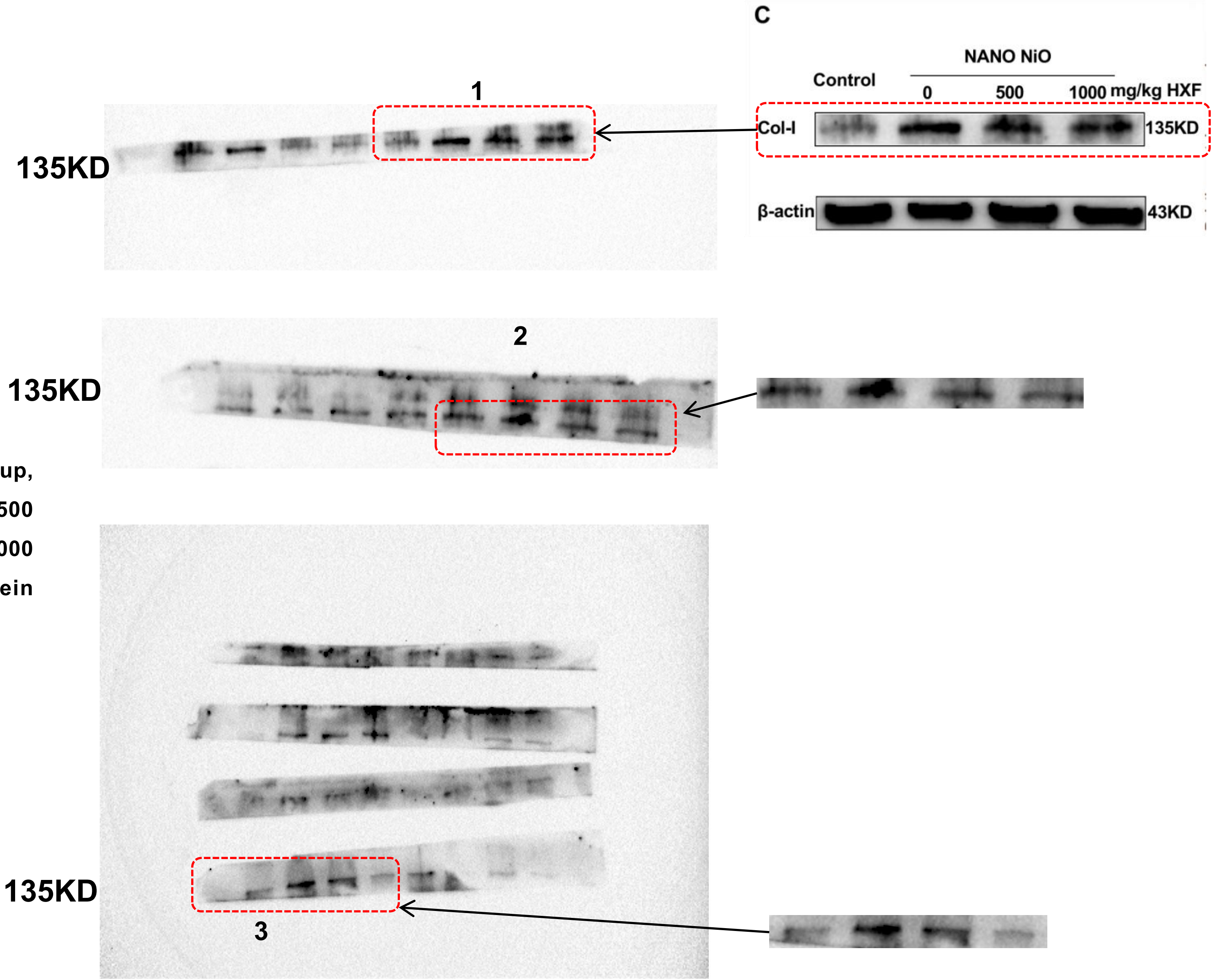

IB

Protein Name: PI3K

Brand: Immunoway

Observed Band: 85KD

Catalog No.: YT6156

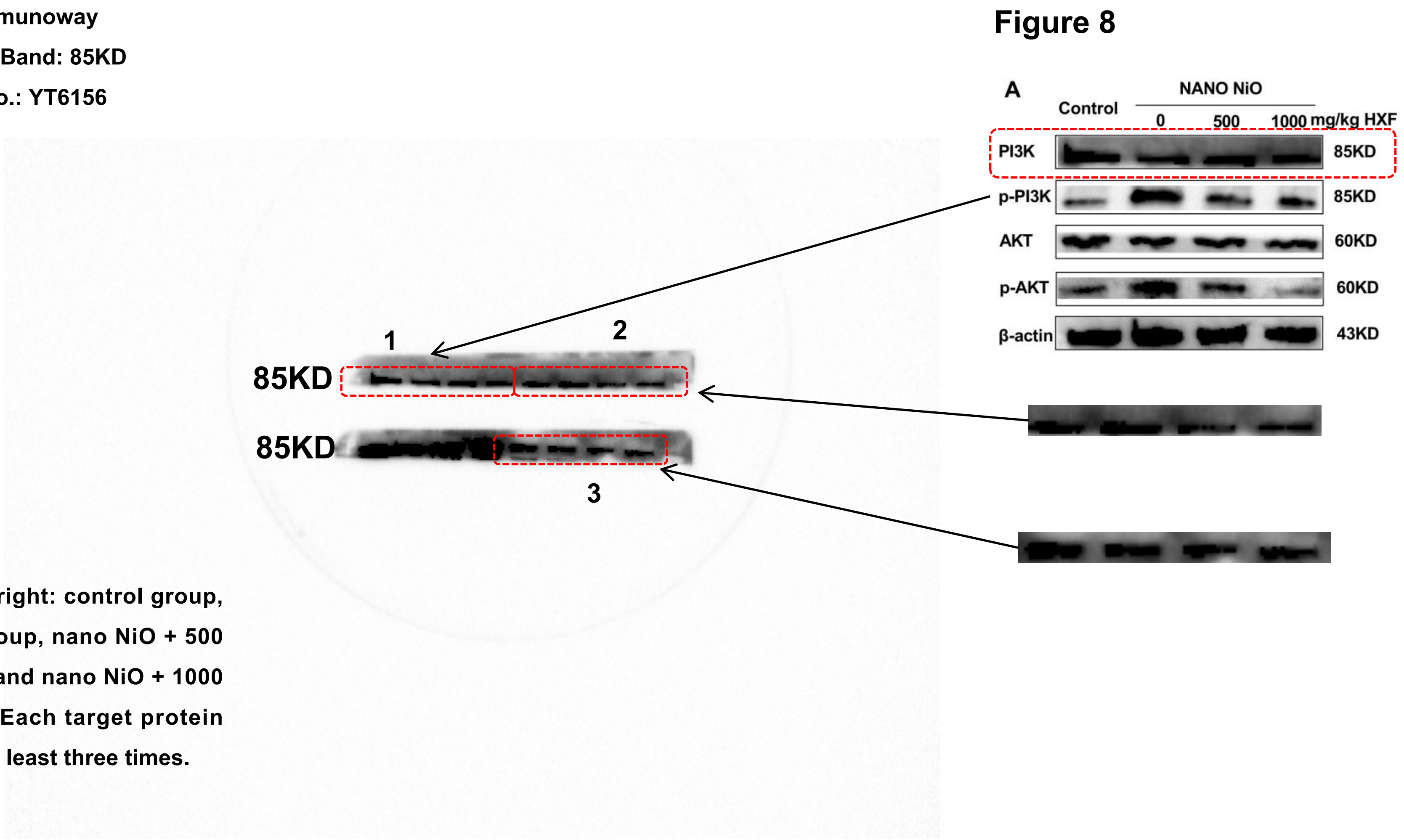

From left to right: control group, nano NiO group, nano NiO + 500 mg/kg HXF, and nano NiO + 1000 mg/kg HXF. Each target protein was tested at least three times.

IB

Protein Name: p-PI3K^{Tyr467}

Brand: Immunoway

Observed Band: 85KD

Catalog No.: YP0224

Figure 8

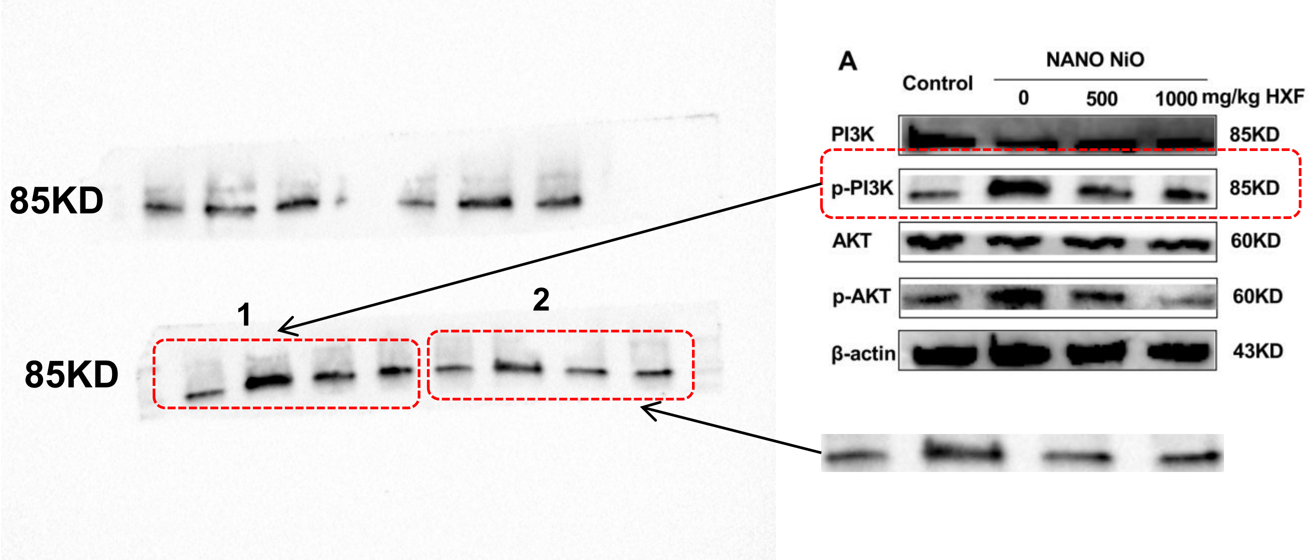

From left to right: control group, nano NiO group, nano NiO + 500 mg/kg HXF, and nano NiO + 1000 mg/kg HXF. Each target protein was tested at least three times.

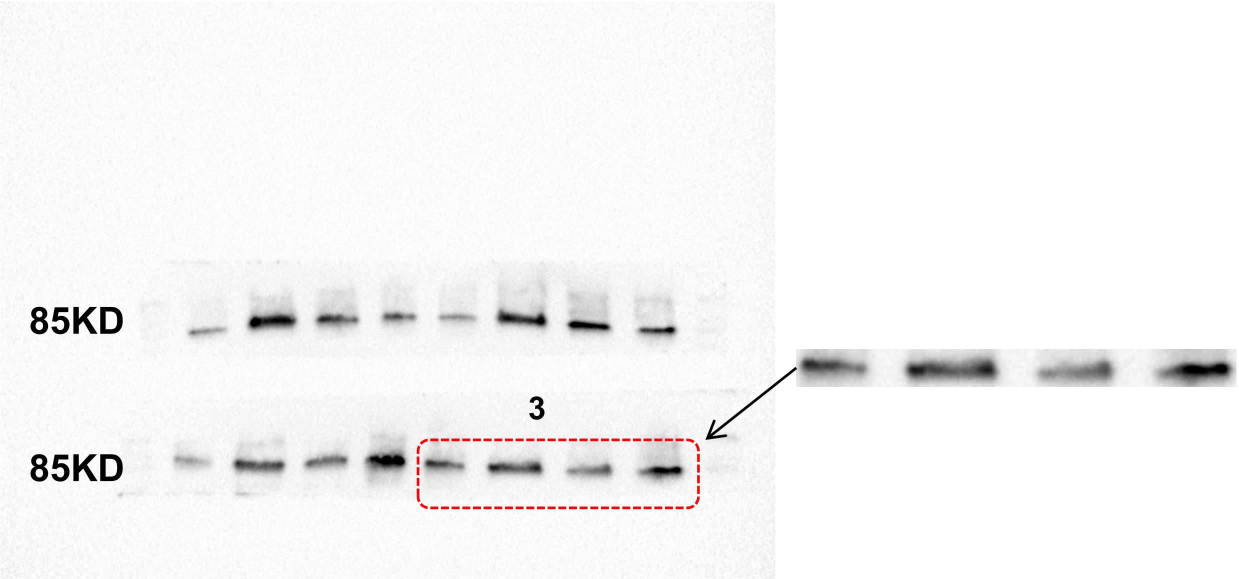

IB

Protein Name: AKT

Brand: Immunoway

Observed Band: 60KD

Catalog No.: YM3618

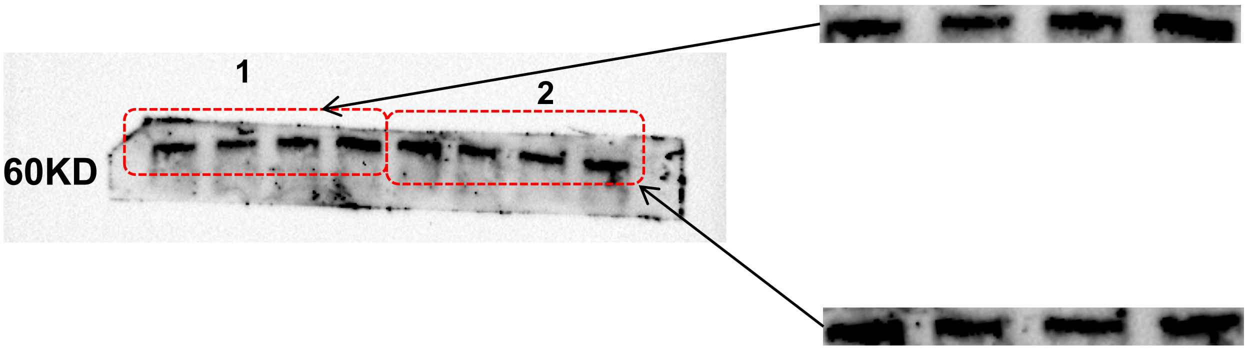

From left to right: control group, nano NiO group, nano NiO + 500 mg/kg HXF, and nano NiO + 1000 mg/kg HXF. Each target protein was tested at least three times.

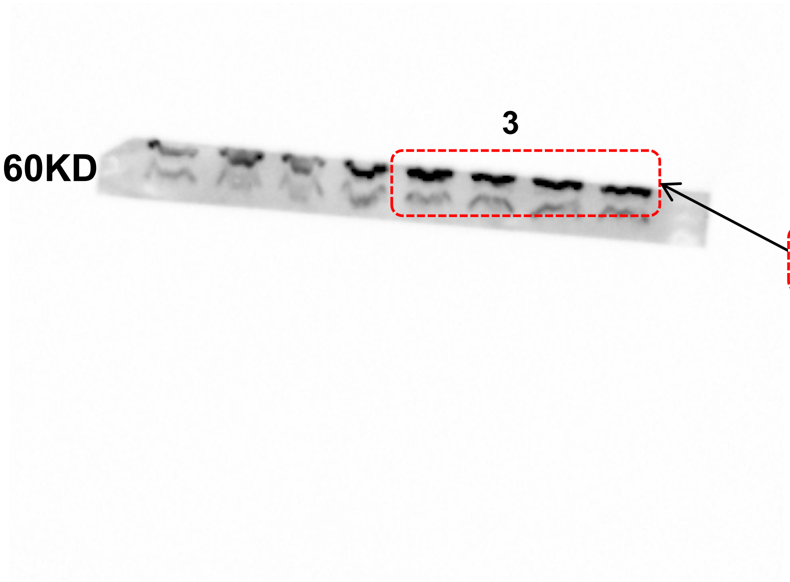

Figure 8

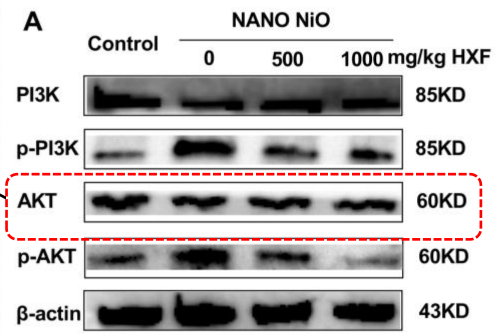

IB

Protein Name: p-AKT^{Ser473}

Brand: Immunoway

Observed Band: 60KD

Catalog No.: YP0006

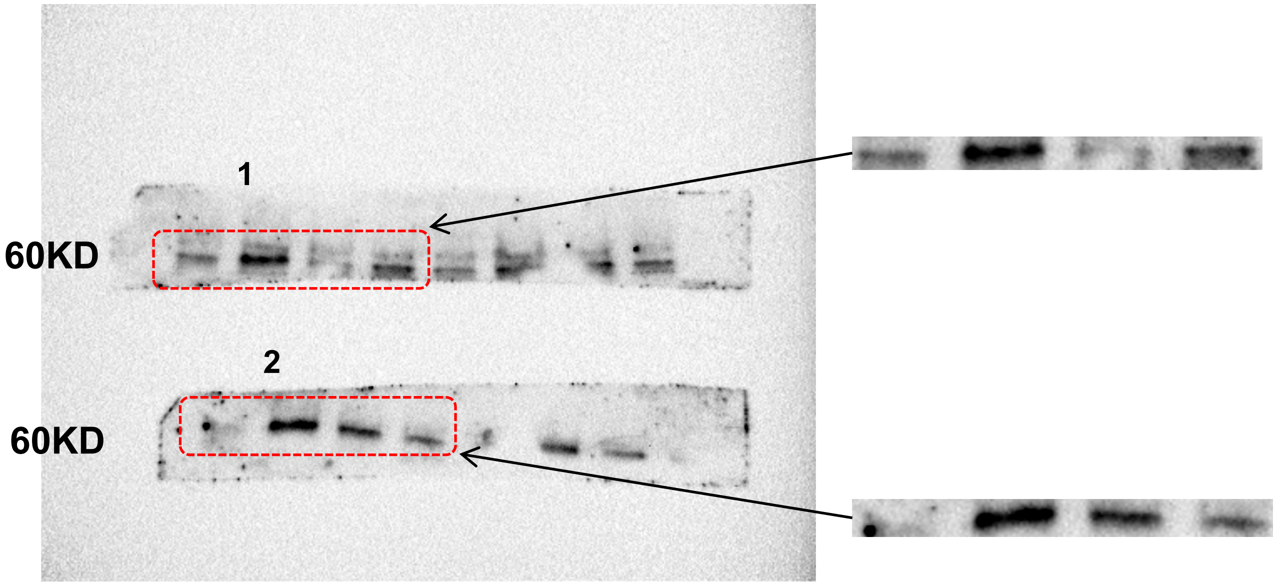

From left to right: control group, nano NiO group, nano NiO + 500 mg/kg HXF, and nano NiO + 1000 mg/kg HXF. Each target protein was tested at least three times.

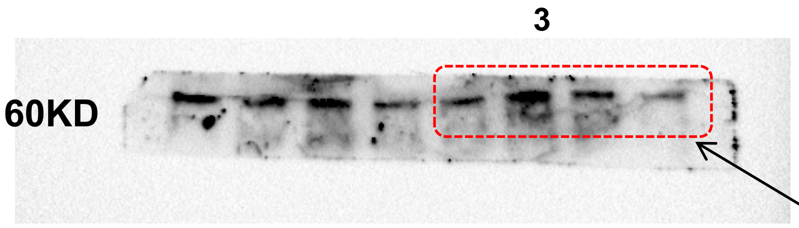

Figure 8

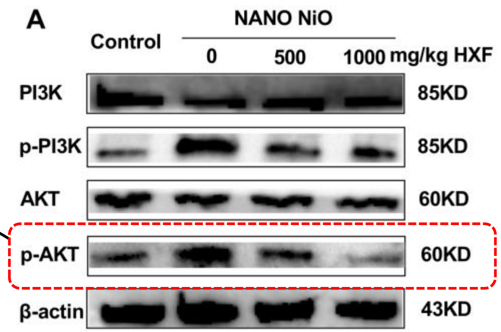

Supplement: Supplementary file 1 — Supplementary Material 1 [file 41598_2025_1899_MOESM1_ESM.pdf]
