# Supplementary material for: Huaxian formula alleviates nickel oxide nanoparticle-induced pulmonary fibrosis via PI3K/AKT signaling
Source: Sci Rep. 2025 May 22;15:17862. doi: 10.1038/s41598-025-01899-y (PMC12098777; doi:10.1038/s41598-025-01899-y)
Supplement: Supplementary file 2 — Supplementary Material 2 [file 41598_2025_1899_MOESM2_ESM.docx]

**Table S1.** 121 bioactive compounds filtered through the ADME system in HXF

| NO. | ID | Compound | OB (%) | DL |
| --- | --- | --- | --- | --- |
| 1 | A1 | kaempferol | 41.88 | 0.24 |
| 2 | B1 | quercetin | 46.43 | 0.28 |
| 3 | C1 | beta-sitosterol | 36.91 | 0.75 |
| 4 | D1 | hederagenin | 36.91 | 0.75 |
| 5 | E1 | Mairin | 55.38 | 0.78 |
| 6 | E2 | Jaranol | 50.83 | 0.29 |
| 7 | F1 | isorhamnetin | 49.60 | 0.31 |
| 8 | H1 | formononetin | 69.67 | 0.21 |
| 9 | H2 | Calycosin | 47.75 | 0.24 |
| 10 | I1 | luteolin | 36.16 | 0.25 |
| 11 | CB1 | Isopimaric acid | 36.20 | 0.28 |
| 12 | CB2 | (5aR,8aS,9R)-9-(3,4,5-trimethoxyphenyl)-5a,6,8a,9-tetrahydro-5H-isobenzofurano[5,6-f][1,3]benzodioxol-8-one | 52.70 | 0.83 |
| 13 | CB3 | DNOP | 40.59 | 0.40 |
| 14 | CB4 | Hinokinin | 56.50 | 0.64 |
| 15 | FJ1 | Hesperetin | 70.31 | 0.27 |
| 16 | FL1 | (2R)-2-[(3S,5R,10S,13R,14R,16R,17R)-3,16-dihydroxy-4,4,10,13,14-pentamethyl-2,3,5,6,12,15,16,17-octahydro-1H-cyclopenta[a]phenanthren-17-yl]-6-methylhept-5-enoic acid | 30.93 | 0.81 |
| 17 | FL2 | trametenolic acid | 38.71 | 0.80 |
| 18 | FL3 | Cerevisterol | 37.96 | 0.77 |
| 19 | FL4 | ergosta-7,22E-dien-3beta-ol | 43.51 | 0.72 |
| 20 | FL5 | Ergosterol peroxide | 40.36 | 0.81 |
| 21 | GC1 | licochalcone a | 40.79 | 0.29 |
| 22 | GC2 | Vestitol | 74.66 | 0.21 |
| 23 | GC3 | Inermine | 75.18 | 0.54 |
| 24 | GC4 | DFV | 32.76 | 0.18 |
| 25 | GC5 | Glycyrol | 90.78 | 0.67 |
| 26 | GC6 | Medicarpin | 49.22 | 0.34 |
| 27 | GC7 | Lupiwighteone | 51.64 | 0.37 |
| 28 | GC8 | 7-Methoxy-2-methyl isoflavone | 42.56 | 0.20 |
| 29 | GC9 | naringenin | 59.29 | 0.21 |
| 39 | GC10 | (2S)-2-[4-hydroxy-3-(3-methylbut-2-enyl)phenyl]-8,8-dimethyl-2,3-dihydropyrano[2,3-f]chromen-4-one | 31.79 | 0.72 |
| 31 | GC11 | euchrenone | 30.29 | 0.57 |
| 32 | GC12 | glyasperin B | 65.22 | 0.44 |
| 33 | GC13 | glyasperin F | 75.84 | 0.54 |
| 34 | GC14 | Glyasperin C | 45.56 | 0.4 |
| 35 | GC15 | Isotrifoliol | 31.94 | 0.42 |
| 36 | GC16 | (E)-1-(2,4-dihydroxyphenyl)-3-(2,2-dimethylchromen-6-yl)prop-2-en-1-one | 39.62 | 0.35 |
| 37 | GC17 | kanzonols W | 50.48 | 0.52 |
| 38 | GC18 | (2S)-6-(2,4-dihydroxyphenyl)-2-(2-hydroxypropan-2-yl)-4-methoxy-2,3-dihydrofuro[3,2-g]chromen-7-one | 60.25 | 0.63 |
| 39 | GC19 | Semilicoisoflavone B | 48.78 | 0.55 |
| 40 | GC20 | Glepidotin A | 44.72 | 0.35 |
| 41 | GC21 | Glepidotin B | 64.46 | 0.34 |
| 42 | GC22 | Phaseolinisoflavan | 32.01 | 0.45 |
| 43 | GC23 | Glypallichalcone | 61.60 | 0.19 |
| 44 | GC24 | 8-(6-hydroxy-2-benzofuranyl)-2,2-dimethyl-5-chromenol | 58.44 | 0.38 |
| 45 | GC25 | Licochalcone B | 76.76 | 0.19 |
| 46 | GC26 | licochalcone G | 49.25 | 0.32 |
| 47 | GC27 | 3-(2,4-dihydroxyphenyl)-8-(1,1-dimethylprop-2-enyl)-7-hydroxy-5-methoxy-coumarin | 59.62 | 0.43 |
| 48 | GC28 | Licoricone | 63.58 | 0.47 |
| 49 | GC29 | Gancaonin A | 51.08 | 0.4 |
| 50 | GC30 | Gancaonin B | 48.79 | 0.45 |
| 51 | GC31 | 3-(3,4-dihydroxyphenyl)-5,7-dihydroxy-8-(3-methylbut-2-enyl)chromone | 66.37 | 0.41 |
| 52 | GC32 | 5,7-dihydroxy-3-(4-methoxyphenyl)-8-(3-methylbut-2-enyl)chromone | 30.49 | 0.41 |
| 53 | GC33 | 2-(3,4-dihydroxyphenyl)-5,7-dihydroxy-6-(3-methylbut-2-enyl)chromone | 44.15 | 0.41 |
| 54 | GC34 | Glycyrin | 52.61 | 0.47 |
| 55 | GC35 | Licocoumarone | 33.21 | 0.36 |
| 56 | GC36 | Licoisoflavone | 41.61 | 0.42 |
| 57 | GC37 | Licoisoflavone B | 38.93 | 0.55 |
| 58 | GC38 | licoisoflavanone | 52.47 | 0.54 |
| 59 | GC39 | shinpterocarpin | 80.30 | 0.73 |
| 60 | GC40 | (E)-3-[3,4-dihydroxy-5-(3-methylbut-2-enyl)phenyl]-1-(2,4-dihydroxyphenyl)prop-2-en-1-one | 46.27 | 0.31 |
| 61 | GC41 | liquiritin | 65.69 | 0.74 |
| 62 | GC42 | licopyranocoumarin | 80.36 | 0.65 |
| 63 | GC43 | Glyzaglabrin | 61.07 | 0.35 |
| 64 | GC44 | Glabridin | 53.25 | 0.47 |
| 65 | GC45 | Glabranin | 52.90 | 0.31 |
| 66 | GC46 | Glabrene | 46.27 | 0.44 |
| 67 | GC47 | Glabrone | 52.51 | 0.5 |
| 68 | GC48 | 1,3-dihydroxy-9-methoxy-6-benzofurano[3,2-c] chromenone | 48.14 | 0.43 |
| 69 | GC49 | 1,3-dihydroxy-8,9-dimethoxy-6-benzofurano[3,2-c] chromenone | 62.90 | 0.53 |
| 70 | GC50 | Eurycarpin A | 43.28 | 0.37 |
| 71 | GC51 | (-)-Medicocarpin | 40.99 | 0.95 |
| 72 | GC52 | Sigmoidin-B | 34.88 | 0.41 |
| 73 | GC53 | (2R)-7-hydroxy-2-(4-hydroxyphenyl)chroman-4-one | 71.12 | 0.18 |
| 74 | GC54 | (2S)-7-hydroxy-2-(4-hydroxyphenyl)-8-(3-methylbut-2-enyl)chroman-4-one | 36.57 | 0.32 |
| 75 | GC55 | Isoglycyrol | 44.70 | 0.84 |
| 76 | GC56 | Isolicoflavonol | 45.17 | 0.42 |
| 77 | GC57 | HMO | 38.37 | 0.21 |
| 78 | GC58 | 1-Methoxyphaseollidin | 69.98 | 0.64 |
| 79 | GC59 | Quercetin der. | 46.45 | 0.33 |
| 80 | GC60 | 3'-Hydroxy-4'-O-Methylglabridin | 43.71 | 0.57 |
| 81 | GC61 | 3'-Methoxyglabridin | 46.16 | 0.57 |
| 82 | GC62 | 2-[(3R)-8,8-dimethyl-3,4-dihydro-2H-pyrano[6,5-f]chromen-3-yl]-5-methoxyphenol | 36.21 | 0.52 |
| 83 | GC63 | Inflacoumarin A | 39.71 | 0.33 |
| 84 | GC64 | icos-5-enoic acid | 30.70 | 0.2 |
| 85 | GC65 | Kanzonol F | 32.47 | 0.89 |
| 86 | GC66 | 6-prenylated eriodictyol | 39.22 | 0.41 |
| 87 | GC67 | 7,2',4'-trihydroxy－5-methoxy-3－arylcoumarin | 83.71 | 0.27 |
| 88 | GC68 | 7-Acetoxy-2-methylisoflavone | 38.92 | 0.26 |
| 89 | GC69 | 8-prenylated eriodictyol | 53.79 | 0.4 |
| 90 | GC70 | gadelaidic acid | 30.70 | 0.2 |
| 91 | GC71 | Gancaonin G | 60.44 | 0.39 |
| 92 | GC72 | Gancaonin H | 50.10 | 0.78 |
| 93 | GC73 | Licoagrocarpin | 58.81 | 0.58 |
| 94 | GC74 | Glyasperins M | 72.67 | 0.59 |
| 95 | GC75 | Glycyrrhiza flavonol A | 41.28 | 0.6 |
| 96 | GC76 | Licoagroisoflavone | 57.28 | 0.49 |
| 97 | GC77 | Odoratin | 49.95 | 0.3 |
| 98 | GC78 | Phaseol | 78.77 | 0.58 |
| 99 | GC79 | Xambioona | 54.85 | 0.87 |
| 100 | GC80 | dehydroglyasperins C | 53.82 | 0.37 |
| 101 | HQ1 | (3S,8S,9S,10R,13R,14S,17R)-10,13-dimethyl-17-[(2R,5S)-5-propan-2-yloctan-2-yl]-2,3,4,7,8,9,11,12,14,15,16,17-dodecahydro-1H-cyclopenta[a]phenanthren-3-ol | 36.23 | 0.78 |
| 102 | HQ2 | 3,9-di-O-methylnissolin | 53.74 | 0.48 |
| 103 | HQ3 | 7-O-methylisomucronulatol | 74.69 | 0.30 |
| 104 | HQ4 | 9,10-dimethoxypterocarpan-3-O-β-D-glucoside | 36.74 | 0.92 |
| 105 | HQ5 | (6aR,11aR)-9,10-dimethoxy-6a,11a-dihydro-6H-benzofurano[3,2-c]chromen-3-ol | 64.26 | 0.42 |
| 106 | HQ6 | Bifendate | 31.10 | 0.67 |
| 107 | HQ7 | FA | 68.96 | 0.71 |
| 108 | HQ8 | 1,7-Dihydroxy-3,9-dimethoxy pterocarpene | 39.05 | 0.48 |
| 109 | JG1 | acacetin | 34.97 | 0.24 |
| 110 | JG2 | Spinasterol | 42.98 | 0.76 |
| 111 | JG3 | cis-Dihydroquercetin | 66.44 | 0.27 |
| 112 | JQM1 | Procyanidin B1 | 67.87 | 0.66 |
| 113 | JQM2 | ent-Epicatechin | 48.96 | 0.24 |
| 114 | JQM3 | (-)-catechin | 49.68 | 0.24 |
| 115 | JQM4 | (+)-catechin | 54.83 | 0.24 |
| 116 | JQM5 | digallate | 61.85 | 0.26 |
| 117 | JQM6 | coumaroyltyramine | 112.90 | 0.20 |
| 118 | JQM7 | Eriodyctiol (flavanone) | 41.35 | 0.24 |
| 119 | JQM8 | (-)-Catechin gallate | 53.57 | 0.75 |
| 120 | JQM9 | 3-methylquercetin | 30.85 | 0.30 |
| 121 | JQM10 | 3,8-dihydroxy-10-methoxy-5-h-isochromeno[4,,3-b]chromen-7-one | 59.70 | 0.49 |

A1 is a shared compound among CB, GC, and HQ; B1 is common to CB, GC, HQ, and JQM; C1 is present in CB, FJ, GC, and JQM; and D1 is a common compound of FL and HQ. Compounds E1, E2, H1, and H2 are shared by GC and HQ; F1 is common among GC, HQ, and JQM; and I1 is found in both JQM and JG.


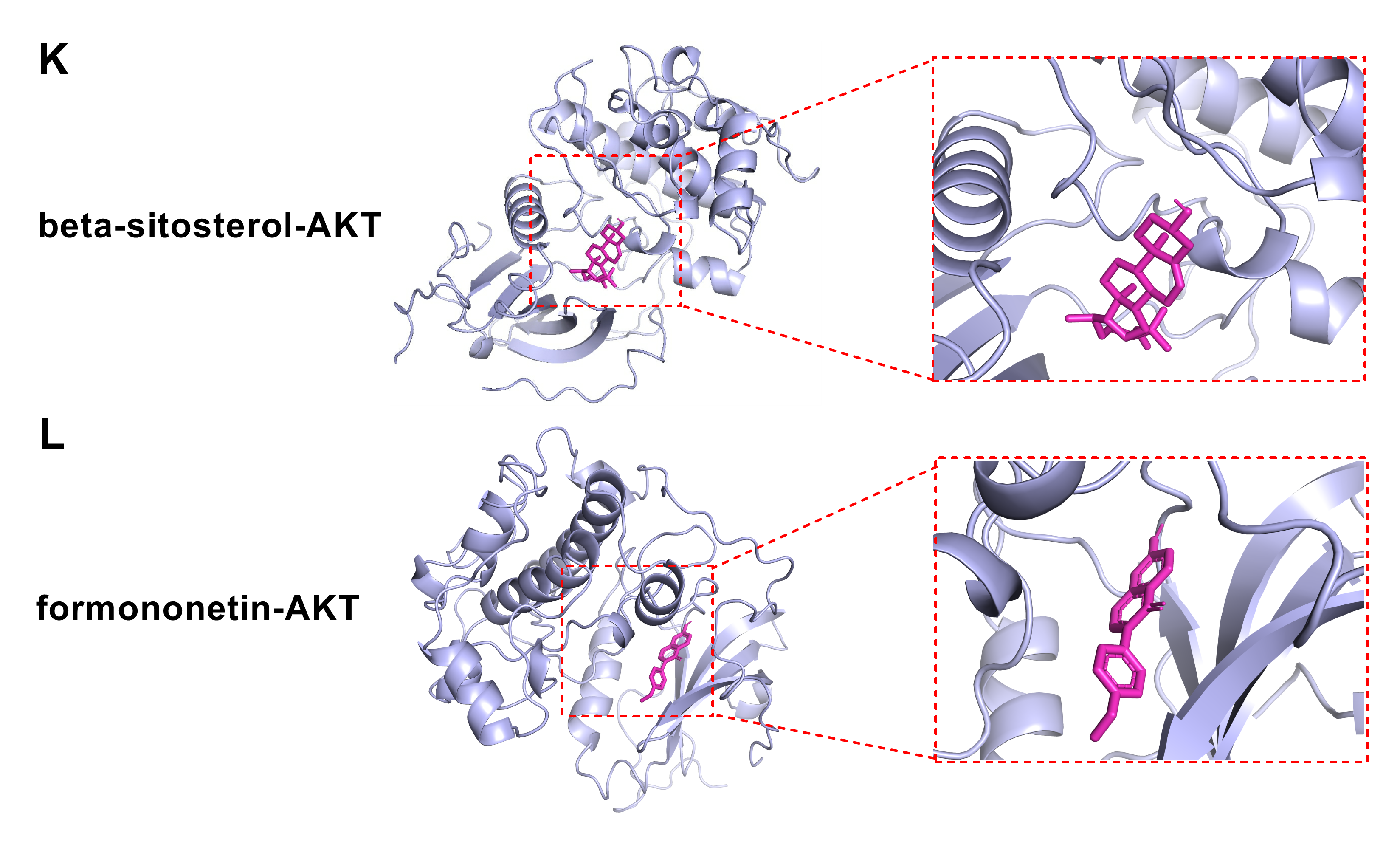


**Figure S1.** The 3D docking patterns and interactions of HXF with the targets in the PI3K/AKT signaling pathway. Beta-sitosterol with AKT and formononetin with AKT did not display polar bonds, implying that their interactions may involve nonpolar forces instead.


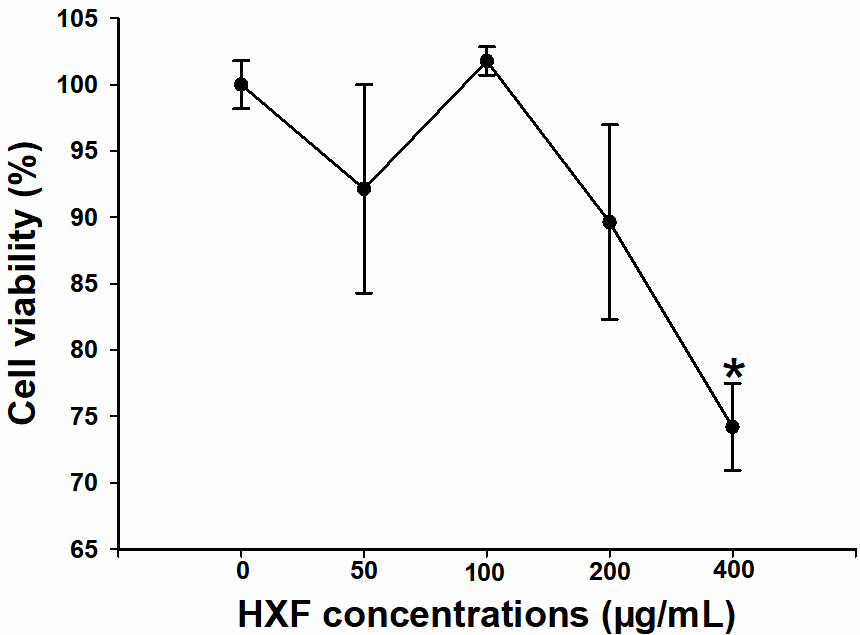


**Figure S2**. Effects of HXF at various concentrations on A549 cell viability (n = 5). *Significant difference compared to the control group (*p* < 0.05).


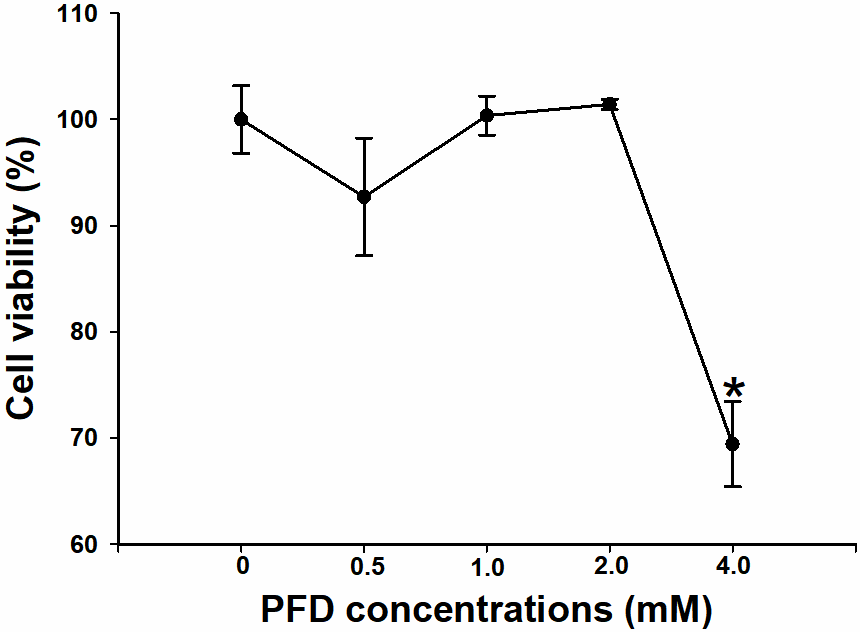


**Figure S3**. Effects of PFD at various concentrations on A549 cell viability (n = 5). *Significant difference compared to the control group (*p* < 0.05).
